# Endothelial CXCR2 deficiency attenuates renal inflammation and glycocalyx shedding through NF-κB signaling in diabetic kidney disease

**DOI:** 10.1186/s12964-024-01565-2

**Published:** 2024-03-25

**Authors:** Siyuan Cui, Xin Chen, Jiayu Li, Wei Wang, Deqi Meng, Shenglong Zhu, Shiwei Shen

**Affiliations:** 1 Department of Endocrinology, The Affiliated Wuxi No.2 People’s Hospital of Nanjing Medical University, Wuxi, China; 2 Department of Endocrinology, Jiangnan University Medical Center, Wuxi, China; 3 Nanjing Medical University, Nanjing, China; 4 Wuxi School of Medicine, Jiangnan University, Wuxi, China

**Keywords:** CXCR2, Diabetic kidney disease, Inflammation, Glycocalyx shedding, NF-κB signaling

## Abstract

**Background:**

The incidence of diabetic kidney disease (DKD) continues to rapidly increase, with limited available treatment options. One of the hallmarks of DKD is persistent inflammation, but the underlying molecular mechanisms of early diabetic kidney injury remain poorly understood. C-X-C chemokine receptor 2 (CXCR2), plays an important role in the progression of inflammation-related vascular diseases and may bridge between glomerular endothelium and persistent inflammation in DKD.

**Methods:**

Multiple methods were employed to assess the expression levels of CXCR2 and its ligands, as well as renal inflammatory response and endothelial glycocalyx shedding in patients with DKD. The effects of CXCR2 on glycocalyx shedding, and persistent renal inflammation was examined in a type 2 diabetic mouse model with *Cxcr2* knockout specifically in endothelial cells (DKD*-Cxcr2* ^eCKO^ mice), as well as in glomerular endothelial cells (GECs), cultured in high glucose conditions.

**Results:**

CXCR2 was associated with early renal decline in DKD patients, and endothelial-specific knockout of CXCR2 significantly improved renal function in DKD mice, reduced inflammatory cell infiltration, and simultaneously decreased the expression of proinflammatory factors and chemokines in renal tissue. In DKD conditions, glycocalyx shedding was suppressed in endothelial *Cxcr2* knockout mice compared to *Cxcr2* ^*L/L*^ mice. Modulating CXCR2 expression also affected high glucose-induced inflammation and glycocalyx shedding in GECs. Mechanistically, CXCR2 deficiency inhibited the activation of NF-κB signaling, thereby regulating inflammation, restoring the endothelial glycocalyx, and alleviating DKD.

**Conclusions:**

Taken together, under DKD conditions, activation of CXCR2 exacerbates inflammation through regulation of the NF-κB pathway, leading to endothelial glycocalyx shedding and deteriorating renal function. Endothelial CXCR2 deficiency has a protective role in inflammation and glycocalyx dysfunction, suggesting its potential as a promising therapeutic target for DKD treatment.

**Supplementary Information:**

The online version contains supplementary material available at 10.1186/s12964-024-01565-2.

## Introduction

Diabetic kidney disease (DKD), which is caused by diabetes-related disorders of glucose metabolism that then trigger other metabolic abnormalities, such as hemodynamic, inflammatory, and fibrotic processes [1], is the primary reason behind the progression of chronic kidney disease (CKD) and the onset of end-stage renal disease (ESRD) [2]. Although strict glycemic and blood pressure control is crucial for managing complications of type 2 diabetes mellitus (T2DM), it has limited effectiveness in delaying the deterioration of renal function in patients with DKD [3–5]. Given the rapid increase in the incidence of DKD and the constrained treatment options, it is crucial to gain a deeper understanding of mechanisms underlying early diabetic kidney injury and to develop targeted drugs for attenuating progressive DKD.

The glomeruli emerge as a pivotal site for early diabetic kidney injury [6]. The glomerular filtration barrier (GFB) consists of the podocyte layer, the glomerular basement membrane (GBM), and the endothelium [7]. Damage to any of these GFB components precipitates albuminuria and glomerular disease. Increasing evidence suggests that glomerular endothelial cells (GECs) sustain injury earlier than podocytes in DKD [8, 9]. Moreover, Weil et al. found that GEC injury was more closely linked to increased urine albumin excretion than podocytes. Interestingly, mitochondrial dysfunction and oxidative stress occur in GECs in the diabetic microenvironment, which in turn compromises neighboring podocytes and contributes to microalbuminuria in the early stages of DKD [6, 9, 10], suggesting the importance of the glomerular endothelium.

Dysfunctional endothelial cells contribute to the progression of the early stage of DKD, resulting in impaired nitric oxide-dependent vasorelaxation, increased vascular permeability, and detachment of leukocytes [11, 12]. Importantly, the endothelial glycocalyx (EG) regulates these functions [13, 14], governing vascular permeability, preventing albumin leakage, and safeguarding endothelial cells from oxidative stress [15–17]. The glycocalyx on the top membrane of GECs is a brush-like polysaccharide-protein complex structure composed of proteoglycans (PGs), glycoproteins, and glycolipids [11, 18]. Heparan sulfate PGs (HSPGs), composed of syndecan and heparan sulfate, are most abundant on the cell surface [19]. In the diabetic microenvironment, inflammation, oxidative stress, and other harmful factors can destroy the glycocalyx directly or indirectly, and then syndecan and heparan sulfate are released into the blood [18, 20, 21]. There exists a vicious circle involving dysfunctional endothelial cells and the glycocalyx, indicating that restoring either component could potentially serve as a therapeutic pathway, especially for early DKD [22].

It is currently believed that diabetes is a chronic inflammatory disease associated with increased levels of inflammatory factors [23–25]. Inflammation plays an important role in glycocalyx shedding directly, and glycocalyx degradation fragments, including heparan sulfate and syndecan, induce inflammation in turn, forming a vicious cycle between inflammation and glycocalyx shedding [1, 22, 26], but the related mechanism is currently not well established. Becker et al. [27] advocated inhibitors of inflammation and inhibitors of metalloproteases as potential means to reduce the degradation of EG in clinical settings, but no inflammatory mediators have been demonstrated to slow DKD progression due to a lack of targeting [23, 27]. While CXCR2, a receptor for the CXC chemokines CXCL1 and CXCL8,

CXCR2, a receptor for the CXC chemokines CXCL1 and CXCL8, plays a critical role in mediating the recruitment and migration of neutrophils and monocytes [28, 29]. IL-8, through its interaction with CXCR2, triggers to the formation of neutrophil extracellular traps that aggravate the progression of atherosclerosis in vivo [29, 30], and CXCR2 was identified as a common hub gene for peripheral arterial disease (PAD) [31]. However, significantly more research has focused on chemokines rather than their receptors, and the precise role and mechanism of CXCR2 in the endothelial cells of DKD are still not fully understood. Based on the above, CXCR2 may be a therapeutic strategy targeting inflammation and glycocalyx shedding in DKD.

In this study, we aimed to evaluate the expression levels of CXCR2 and its ligands in DKD and determine their correlation with renal function. We also investigated the specific role and mechanism of CXCR2 in the progression of glycocalyx shedding associated with inflammation in mice with endothelial knockout of *Cxcr2*. Additionally, we studied the detailed mechanisms by which CXCR2 mediates glycocalyx shedding through high glucose-induced GECs. The results demonstrate that CXCR2 exacerbates the inflammatory response and glycocalyx dysfunction in DKD by activating the NF-κB signaling pathway. Targeted inhibition of CXCR2 in specific tissues is likely to be a potential therapeutic strategy for DKD.

## Methods

### Blood and kidney sample collection

A research study is ongoing from January 2021 to April 2022. It aims to involve 50 patients with type 2 diabetes (T2DM) and a control group of 50 healthy individuals, all treated at the Endocrinology Department of the Jiangnan University Medical Center (JUMC). Among the T2DM patients, 50 met the diagnostic criteria for diabetic kidney disease (DKD), which is characterized by a urinary albumin-to-creatinine ratio (UACR) greater than 30 mg/g and an estimated glomerular filtration rate (eGFR) less than 60 mL/min/1.73 m^2^. General information and clinical data were collected from the electronic medical records, including age, sex, body mass index (BMI), fasting plasma glucose (FPG), glycated hemoglobin (HbA1c), blood urea nitrogen (BUN), serum creatinine (Scr), estimated glomerular filtration rate (eGFR), urine albumin creatinine ratio (UACR), α1-microglobulin (α1-MG), and retinol-binding protein (RBP), detailed in Tab. S1. Biochemical indicators were analyzed using an automatic biochemical analyzer (Beckman, AU5800). Additionally, kidney biopsies were obtained from 10 DKD patients at the Nephrology Department of JUMC. Kidneys from the control group were sourced from nondiabetic patients undergoing renal surgery for chronic kidney cancer at JUMC, with samples taken from tumor-free kidney areas. Exclusion criteria included age < 18 years, presence of other kidney diseases, pregnancy, infection, and genetic diseases. The study was approved by the Ethics Committee of JUMC (Ethics Review No. Y-125), and all participants provided informed consent.

### Animal experimentation

The mice were housed in a specific pathogen-free facility with a 12-hour light-dark cycle and ad- libitum access to standard food. Endothelial cell-specific *Cxcr2* knockout mice (C57BL/6 N-*Cxcr2* ^em1cyagen^, Serial Number: CKOCMP-12765-*Cxcr2*-B6N-V, *Cxcr2* ^eCKO^) were purchased from Cyagen Biosciences Inc.(Suzhou, China), and *Cxcr2* ^L/L^ represents littermate control for *Cxcr2* ^eCKO^. The validation process of *Cxcr2* ^eCKO^ mice was presented in Fig. S2A-B, Which showed a high efficiency in Fig. S2D-F. The experiment was divided into four groups: *Cxcr2* ^L/L^ group, *Cxcr2* ^eCKO^ group, DKD-*Cxcr2* ^L/L^ group, and DKD-*Cxcr2* ^eCKO^ group. To generate a model of T2DM, 8-week-old *Cxcr2* ^L/L^ and *Cxcr2* ^eCKO^ mice were fed a high-fat diet (including 20% sucrose, 10% lard, and 3% cholesterol), followed by intraperitoneal injection of 50 mg/kg STZ (Sigma, S0130) for 5 consecutive days. Meanwhile, the control mice were fed a chow diet injected with citrate buffer. After 3 days, the blood glucose levels of the mice were measured using a Roche glucose analyzer, and if the blood glucose level exceeds 16.7 mmol/L, it indicates successful modeling of diabetic mice. Body weight and blood glucose levels were monitored biweekly. Glucose tolerance tests (GTTs) were conducted, and urine samples were collected using metabolic cages. The urinary levels of microalbumin (MAU) and creatinine were measured using assay kits (MIBio, ml025061, ml026283), and UACR was calculated based on the ratio of MAU to creatinine. Renal function of mice were also assayed by serum creatinine (Scr) and blood urea nitrogen (BUN) using the corresponding kits (Nanjing Jiancheng Bioengineering Institute, China).

### Plasma triglyceride (TG) and plasma cholesterol (TC) measurement

The test kits for plasma TG and TC of mice (Nanjing Jian Cheng Bioengineering Institute, China) were equilibrated to room temperature. The blank wells were supplemented with sample dilution buffer, while the remaining wells were supplemented with standard or test samples. The plate was then incubated at 37 °C for 20 minutes. Next, working solution A was added to each well and incubated at 37 °C for 60 minutes. Then, the liquid was discarded, and the plate was washed three time. Then, working solution B was added and incubated for another 60 minutes. Subsequently, the substrate solution was added and incubated in a light-protected environment for 5 minutes. Finally, the stop solution was rapidly added, and the optical density (OD) was sequentially measured at a wavelength of 450 nm.

### Isolation of mice glomeruli and MGECs

Mice were deeply anesthetized with isoflurane and perfused with saline. Subsequently, a midline incision was made to expose the abdominal cavity, and both kidneys were rapidly extracted. The outer capsule of the kidneys, perirenal fat tissue, and renal medulla were removed using a scalpel blade. The remaining renal cortex was minced and ground. Subsequently, the renal cortex was sequentially filtered through metal mesh filters with pore sizes of 180 μm, 100 μm, 74 μm, and 50 μm. The renal glomeruli retained on the 50 μm filter were collected in a 50 ml centrifuge tube. The tissue was then centrifuged at 1800x g for 5 minutes, and the pellet was gently resuspended in ice-cold HBSS. The number of renal glomeruli was observed under a microscope in the resuspended solution. After two washes with HBSS, the glomeruli were treated with collagenase I (Sigma, C9891, 2 mg/ml), DNAse I (Sigma, D4527, 27 IU/ml) for 30 minutes at 37 °C. During the digestion period, the solution was vortexed every 10 minutes, and then was centrifuged for 6 minutes at 400×g, and resuspended in HBSS [32, 33]. The mouse glomerular endothelial cells (MGECs) were further enriched using mouse CD31 magnetic microbeads (Miltenyi, Biotec130097418) according to the manufacturer’s protocol, MGECs were identified using CD31 immunofluorescence staining before used for experiments.

### Cell culture experimentation

GECs were purchased from ScienCell Company (San Diego, CA, USA), and cultured in DMEM/F12 medium containing 10% fetal bovine serum (FBS; VivaCell Biotechnology, C04001) at 37 °C in a humidified atmosphere of 5% CO_2_. After overnight starvation, GECs were pretreated with high glucose (HG, 30 mM) for 48 h. Glucose (5 mM) plus mannitol (25 mM) was used as an osmotic control in this experiment. Either 100 ng/ml LPS (Lipopolysaccharides, Sigma, L2880) was added as an activator, or 10 μM BAY11–7082 (MedChemExpress, HY-13453) was added as an inhibitor of NF-κB signaling to the culture medium.

### Small interfering RNA (siRNA) transfection or plasmid transfection

CXCR2 siRNAs, designed and synthesized by Shanghai Talen-bio Scientific Co. Ltd. were added at a concentration of 50 nM for 48 h to effectively knockdown the expression of CXCR2. The mRNA and protein expression of CXCR2 in GECs was detected after transfection with CXCR2 siRNAs. The nucleotide sequences of CXCR2 siRNAs are detailed in Tab. S2. The universal negative control siRNA was used as a control. The pcDNA3.1-CXCR2 was added for CXCR2 overexpression in GECs, and the pcDNA3.1 empty vector was used as a control. After transfection with pcDNA3.1-CXCR2 or pcDNA3.1 empty vector for 4 hours, the medium containing the transfection reagents was replaced with complete medium. Transfection efficiency was assayed after 48 hours of incubation. Transfection for CXCR2 siRNAs or pcDNA3.1-CXCR2 was carried out with jetPRIME (Polyplus, 101,000,046) reagent according to the manufacturer’s protocol individually. To eliminate the influence of osmotic pressure on this experiment, the control group served as a mannitol control group and negativel siRNA or pcDNA3.1 empty vector was added in control group and HG group during transfection to ensure consistency in experimental conditions.

### ELISA measurement

The concentrations of the inflammatory cytokines CXCL1, CXCL8, TNF-α, IL-1β, IL-6, and IL-18, and the levels of syndecan-1, syndecan-4, and heparan sulfate were determined by ELISA according to the manufacturer’s instructions, human: CXCL1 (MIBio, YJ057794), CXCL8 (MIBio, YJ027375), syndecan-1 (MIBio, YJ027595), syndecan-4 (LABio, JL11454), and heparan sulfate (MIBio, YJ922620); mouse: TNF-α(MIBio, ml002095), IL-1β (MIBio, ml001814), IL-6 (MIBio, ml002293), IL-18 (MIBio, ml002294), syndecan-1 (Cloud-Clone Corp, SEB966Mu), and heparan sulfate (Cloud-Clone Corp, SEA178Mu).

### Special staining and immunohistochemistry

Paraffin sections (4 μm) of kidneys stained with hematoxylin and eosin (HE) and periodic acid–Schiff (PAS) were used for the evaluation of kidney histology. After deparaffinization with xylene, dehydration with alcohol, antigen retrieval with sodium citrate solution, and blocking with 3% H_2_O_2_, the sections of renal tissue were incubated with primary antibodies CXCR2 (1:250, Proteintech, 20,634–1-AP), F4/80 (1:200, Proteintech, 29,414–1-AP), MPO (1:100, Biovision, 3831–100), E-selectin (1:200, Santa Cruz, sc-137,054), syndecan-1 (1:500, Abcam, ab128936), and syndecan-4 (1:200, Proteintech, 11,820–1-AP) at 4 °C overnight. After washing with PBS, the sections were incubated with secondary antibodies for 30 minutes. Quantification of mesangial expansion was performed using PAS staining, which was characterized as a periodic acid–schiff-positive area devoid of nuclei within the mesangium. Images were scanned, and the profile areas of glomeruli were outlined using ImageJ (NIH, USA) under 400x magnification (Leica SP8 confocal microscope). The glomerular cross-sectional area was calculated with HE staining [34, 35]. Additionally, ImageJ software was employed to assess the integrated optical density (IOD) value of the IHC section in glomeruli. The 400x images were converted to 8-bit grayscale. Background density was measured by selecting three distinct unstained areas in the background by ImageJ to eliminate any influence of background color on positive expression analysis, termed as “threshold”. The Glomerular regions were then selected for measurement of area and IOD [36, 37]. Finally, the mean integrated optical density was calculated as IOD divided by the selected glomerular area. For all special staining and immunohistochemistry above, randonly 15 glomeruli (three samples for each group and five randomly glomeruli of each sample were evaluated) were selected per group to analyze and subjected to statistical analysis. Furthermore, all sections were analyzed with the guidance of professionals in our pathology department in a blinded manner.

### Immunofluorescence

Mice were deeply anesthetized with isoflurane, perfused with saline and ice-cold 4% paraformaldehyde. Kidney tissues were fixed with 4% paraformaldehyde for 24 h and cryoprotected in 30% sucrose at 4 °C for 1 week. Frozen sections of kidneys were prepared for 25 μm, and the sections were washed three times with PBS for 5 minutes. Then, 5% bovine serum albumin (BSA, Beyotime Biotech) was used for blocking for 2 h. The sections were stained with the following primary antibodies: CXCR2 (1:200), Syndecan-1 (1:300), E-selectin (1:100) and heparan sulfate (1:300, Abcam, ab2501) at 4 °C overnight. After washing with PBS, the sections were incubated with goat anti-rabbit IgG H&L (Alexa Fluor® 488, Abcam, ab150077), and goat anti-mouse IgG H&L (Alexa Fluor® 594, Abcam, ab150116) for 2 h at 37 °C. GECs were observed at 200x magnification. All immunofluorescence images of mice were captured using a confocal laser scanning microscope (× 630, Zeiss, LSM 880). Image J software was utilized to measure the mean intensity of immunofluorescence in the glomeruli. Initially, the images were converted to 8-bit grayscale, and a threshold was selected. Glomerular regions were then delineated for the measurement of parameters such as area, integrated density, mean gray value, etc., as described in the referenced article [38]. Five glomeruli per mouse (six mouse for each group) were randomly selected and measured in a blinded manner, followed by statistical analysis.

### Electron microscopy

The mice were deeply anesthetized with isoflurane, following which they were perfused with a 2% lanthanum nitrate (Macklin, 10,277,437) solution through the left ventricle until the kidneys appeared white. Subsequently, they were perfused with a fixative solution consisting of 2% lanthanum nitrate (Macklin, 10,277,437), 2% paraformaldehyde, and 2.5% glutaraldehyde (Macklin, 111,308). Kidney tissues were then collected and cut into approximately 1 mm^3^-sized pieces [39]. After being placed in the fixative solution for 24 hours at 4 °C. After washing, tissues were dehydrated through graded acetone (30,50,70,80,90,100, 100%, 8 min each at 4 °C). Samples were infiltrated in graded mixture (3:1, 1:1, 1:3) of acetone and SPI-PON812 resin (21 ml SPI-PON812,13 ml DDSA and 12 ml NMA), then changed pure resin. Finally, tissues were embedded in pure resin with 1.5% BDMA and polymerized for 12 h at 45 °C, 48 h at 60 °C. The ultrathin sections (70 nm thick) were sectioned with microtome (Leica EM UC6), double-stained by uranyl acetate and lead citrate, and examined by a transmission electron microscope (FEI Tecnai Spirit120kV) with the EMSIS CCD camera (VELETA).

EG depth and coverage were quantified using transmission electron microscopy. Electron micrographs were captured using a transmission electron microscope (FEI Tecnai Spirit120kV) with the EMSIS CCD camera (VELETA), and image analysis was conducted on 3 capillary loops per glomerulus and 5 glomeruli per mouse (three mouse for each group) [21, 40]. In summary, ImageJ software was utilized to overlay a grid onto the electron micrograph. The anatomical distance from the luminal phospholipid at sequential grid intersections to the furthest point of the glycocalyx was measured as glycocalyx depth. A glycocalyx depth of ≤10 nm was considered as uncovered, and this value was expressed as a percentage of the total measurements taken on a grid section. Glycocalyx coverage, expressed as a percentage, was calculated as 100% minus the percentage of uncovered glycocalyx. Observers were blinded to sample identity before analysis.

### RNA isolation and qPCR

For qPCR analysis, total mRNA was extracted from glomeruli or cell samples using the Ultrapure RNA Kit (CWBIO, CW0581M). RNA samples were reverse-transcribed using HiScript III All-in-one RT SuperMix Perfect for qPCR (Vazyme Biotech, R333–01). The assay was performed on a Roche Light Cycler 480 II system using Hieff® qPCR SYBR Green Master Mix (Yeasen Biotech, 11201ES03). The cycling conditions comprised 40 cycles of denaturation at 95 °C for 15 s and annealing/extension at 60 °C for 30 s. Results were normalized to the housekeeping gene (β-actin) and presented as 2^−ΔΔCt^. Primer sequences were synthesized by Invitrogen and listed in Tab. S3 and Tab. S4.

### Western blot

Total protein from glomeruli or cell samples was extracted using RIPA buffer (Beyotime Biotechnology, P0013B) containing protease inhibitor and phosphatase inhibitor. Equal amounts of protein were separated by sodium dodecyl sulfate–polyacrylamide gel electrophoresis (SDS–PAGE) and then transferred to a polyvinylidene fluoride (PVDF) membrane (Millipore, ISEQ00010). After blocking the membrane with 5% skim milk in TBST, the PVDF membrane was incubated with primary antibodies: p-IKKβ (1:1000, Cell Signaling Technology, 2697 T), IKKβ (1:1000, CST, 2678 T), p-IκBα (1:1000, CST, 2859), IκBα (1:1000, CST, 4812S), p-NF-κB p65 (1:1000, CST, 3033P), NF-κB p65 (1:1000, CST, 8242S), CXCR2 (1:1000, Bioss, bs1629R) and β-actin (1:5000, ABclonal, AC026). After incubation overnight at 4 °C and incubated with secondary antibodies: anti-rabbit IgG conjugated to horseradish peroxidase (HRP) and anti-mouse IgG conjugated to HRP for 2 h at room temperature. Protein visualization was performed using an ECL chemiluminescence detection kit (Biotanon Biotechnology, China), and protein density was analyzed using ImageJ software. Throughout the experiment, β-actin was used as an internal reference.

### Statistical methods

Statistical analysis was performed using GraphPad Prism 8.0 and SPSS 25.0 software (SPSS Inc., Chicago, IL, USA). Data are presented as the mean ± standard error of the mean (SEM). A t test or Mann–Whitney nonparametric test was employed for comparing variables between two groups. One-way ANOVA or Dunn’s posttest were utilized for multiple groups. Line regression was used to assess the correlation between UACR and the expression of the chemoattractant factors, syndecan-1. A significance level of *P* < 0.05 was considered statistically significant.

## Results

### Elevated CXCR2 expression correlates with glycocalyx damage during the progression of DKD

**Fig. 1:**
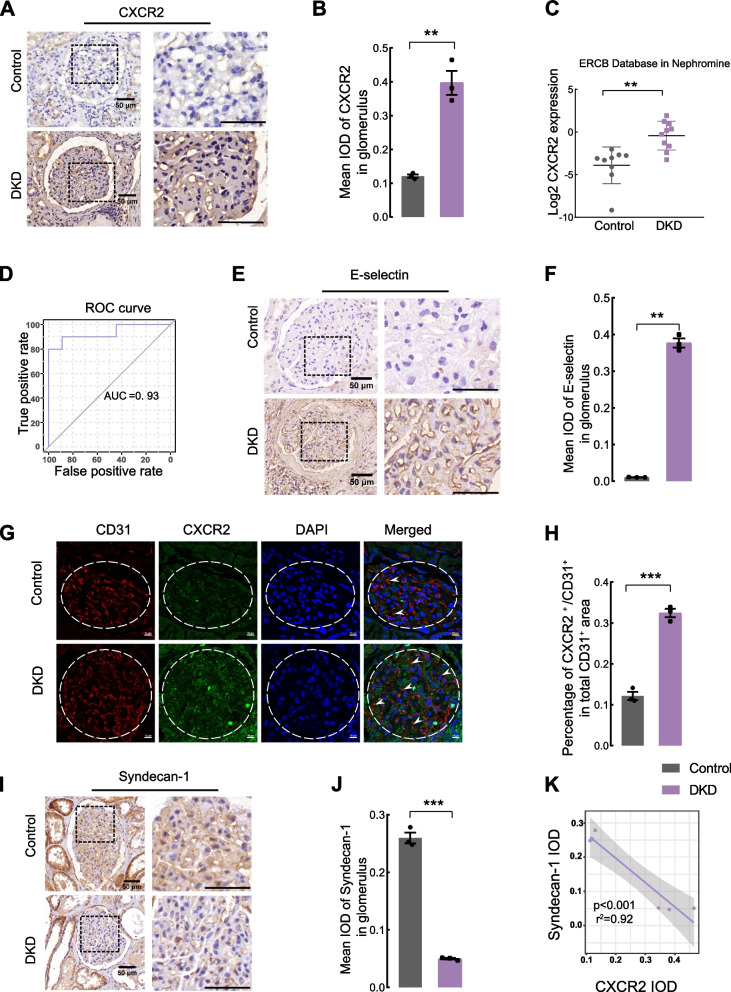
CXCR2 level is increased in patients with DKD and correlated with glycocalyx damage. Immunohistochemical staining (**A**) and scores (**B**) for the expression of CXCR2 are shown in glomeruli and enlarged glomerular image of the box (Scale bar = 50 μm, *n* = 3 for each group).(**C**) The mRNA level of CXCR2 in DKD and healthy controls was obtained from www.v5.nephroseq.org of ERCB Database. **D** A receiver operating characteristic (ROC) curve was used to assess the diagnostic value of CXCR2 for DKD patients based on the data of mRNA level of ERCB Database. Immunohistochemical analysis (**E**) and quantification (**F**) of E-selection expression in glomeruli and enlarged glomerular image of the box,positive nuclear staining for E-selection was localized in endocapillary area of enlarged glomerular (Scale bar = 50 μm, *n* = 3 for each group). Immunofluorescence colocalization (**G**) and analysis (**H**) of CXCR2 and CD31 in the glomeruli of human (Scale bar = 10 μm, *n* = 3, glomeruli were outlined with dotted lines). Immunohistochemical staining (**I**) and scores (**J**) for syndecan-1 in glomeruli and enlarged glomerular image of the box (Scale bar = 50 μm, *n* = 3 for each group). **K** Line regression was used to assess the correlation between CXCR2 expression and syndecan-1 expression of glomeruli based on the data of IOD in DKD patients and controls (*p* < 0.001,r^2^ = 0.92). The images for immuohistochemistry:× 630, the images for immunofluorescence:× 400. Error bars indicate SEM, and data represent mean ± SEM. ^**^P < 0.01, ^***^P < 0.001 vs. control group; IOD,integrated optical density; r^2^, the correlation coefficient; DKD, diabetic kidney disease; UACR, urine albumin creatine ratio; ROC, receiver operating characteristic; AUC, area under the curve

The clinical information and specimens were collected from healthy individuals and DKD patients (Tab. S1, *P* ^**^ < 0.01). The expression of CXCR2 in glomeruli was significantly increased in DKD patients compared to controls by immunocytochemistry (Fig. 1A, B). Meanwhile, CXCR2 mRNA level was increased in kidneys of DKD patients (Fig. 1C, from the www.v5.nephroseq.org ERCB database). A receiver operating characteristic (ROC) curve suggested that CXCR2 might serve as a diagnostic marker for DKD (Fig. 1D). Interestingly, we observed a significant increase in E-selectin expression within the glomeruli of DKD patients (Fig. 1E, F). We further validated an increase in CXCR2 expression in GECs (marked by CD31) (Fig. 1G, H). The serum levels of the CXCR2 ligands*,* CXCL1 and CXCL8, were also significantly increased in DKD patients (Fig. S1A, S1C), and line regression showed that CXCL1 (Fig. S1B) and CXCL8 (Fig. S1D) were positively correlated with UACR. Furthermore, DKD patients exhibited decreased levels of glycocalyx markers, syndecan-1 (Fig. 1I, J), and syndecan-4 (Fig. S1HI) in the glomeruli compared with controls. We also observed a significant association between higher serum syndecan-1 level (Fig. S1E) and elevated UACR (Fig. S1F), and syndecan-4 level was increased in the circulation of DKD patients (Fig. S1G). Finally, the line regression analysis showed a negative correlation between kidney CXCR2 expression and syndecan-1 expression (Fig. 1K).

### Deletion of Cxcr2 in glomerular endothelium markedly improves renal function in DKD mice

**Fig. 2:**
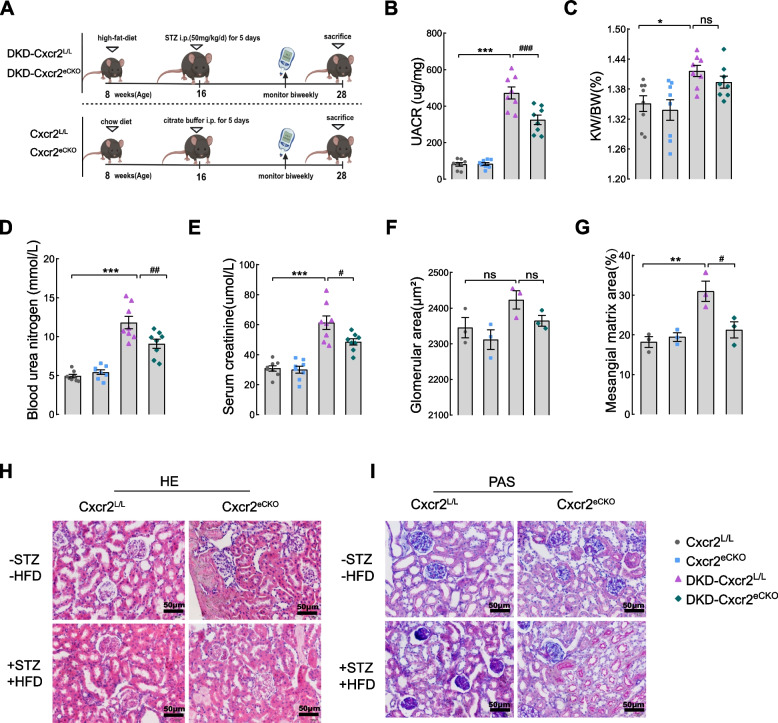
Glomerular endothelial CXCR2 knockout restores renal function in DKD mice. **A** Schematic of the experiment. Kidney-related indicators included (**B**) the urine albumin-to-creatinine ratio (UACR), **(C)** Kidney weight/body weight (KW/BW), (**D**) blood urea nitrogen (BUN), and (**E**) serum creatinine (Scr) were measured (*n* = 8 per group)**.** Quantification of cross-sectional area of glomerular with hematoxylin and eosin (HE). **F** Periodic acid-Schiff (PAS)-stain was used for quantifing mesangial matrix fraction (**G**).** H** Representative images of HE staining in kidneys. **I** Representative images of PAS-stained kidneys (× 400; Scale bar = 50 μm, *n* = 3 mice,five randomly glomeruli were selected per mouse). Results are expressed as mean ± SEM; *P < 0.05, **P < 0.01, and ***P < 0.001 vs. CXCR2 ^L/L^ group; ^#^ *P* < 0.05,^##^ *P* < 0.01 and ^###^ *P* < 0.001 vs. DKD-CXCR2 ^L/L^ group, ^ns^ *P* > 0.05)

As shown in Fig. 2 and Fig. S2, there were no significant differences in any of the indices between *Cxcr2* ^L/L^ and *Cxcr2* ^eCKO^ mice fed a chow diet. Elevated levels of UACR, BUN, and Scr in DKD mice were markedly reduced by endothelial *Cxcr2* knockout (Fig. 2B and D-E), demonstrating that endothelial cell-specific *Cxcr2* knockout could inhibit renal dysfunction in DKD mice. However, the ratio of KW/BW (Fig. 2C), glomerular area (Fig. 2F and H), blood glucose level, body weight, TC and TG (Fig. S2G-J) showed no statistically significant difference between DKD-*Cxcr2* ^eCKO^ and DKD-*Cxcr2* ^L/L^ mice. We found reduced mesangial matrix expansion in DKD-*Cxcr2* ^eCKO^ mice as evidenced by PAS staining (Fig. 2G and I), suggesting that the deterioration of kidney structure in DKD mice is also significantly improved after endothelial deletion of *Cxcr2*.

### Endothelial Cxcr2 knockout obviously suppresses renal glycocalyx shedding and inflammation

**Fig. 3:**
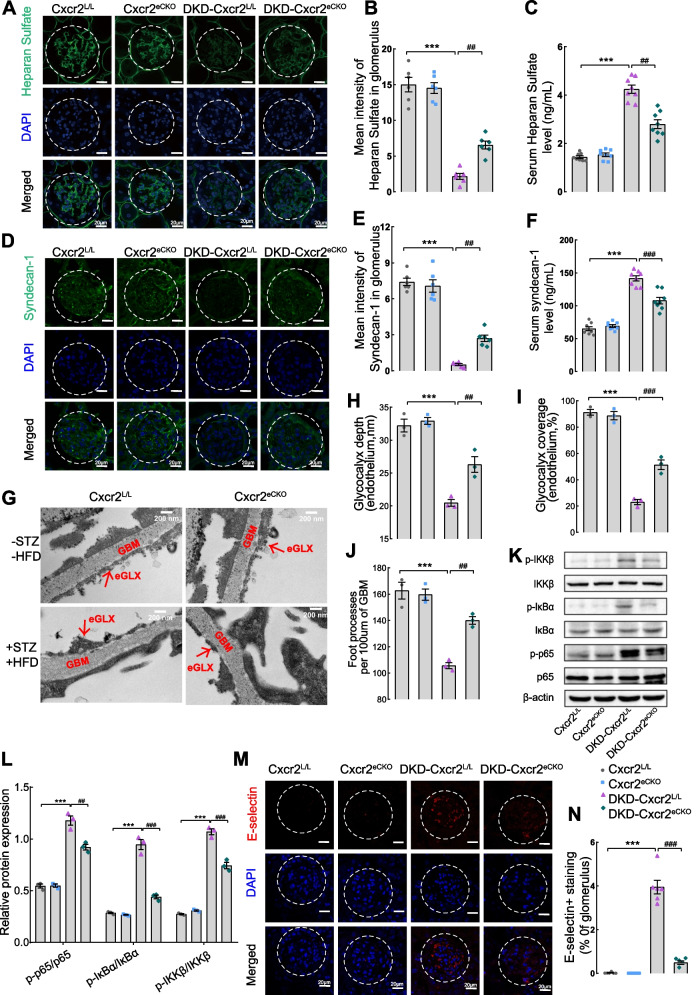
Glycocalyx shedding and the NF-κB signaling pathway in the four groups of mice. Representative images of immunofluorescence staining and corresponding scores were used to analyze the expression of heparan sulfate (**A** and **B**) and syndecan-1 (**D** and **E**) in the glomeruli of the four groups (× 630, Scale bar = 10 μm, *n* = 6 per group, glomeruli were outlined with dotted lines, five randomly glomeruli were selected per mouse). ELISA was used to measure the heparan sulfate level (**C**) and syndecan-1 (**F**) level in the serum of mice (*n* = 8 per group). **G** Electron micrographs were mcaptured to observe the glycocalyx more intuitively (scale bar = 200 nm). Representative electron micrographs of the glomerular capillary wall were shown. The measurements were carried out on 3 capillary loops per glomerulus and 5 glomeruli were used per mouse. Red labels indicate endothelial glycocalyx (eGLX),and glomerular basement membrane (GBM). Quantification of eGLX depth (**H**), percentage endothelium with GLX coverage (**I**) and foot processes (**J**)(*n* = 3 per group)**.** The protein levels of p-IKKβ, IKKβ, p-IκBα, IκBα, p-NF-κBp65, and NF-κBp65 in the glomeruli of mice were determined by western blotting (**K** and **L**) (*n* = 3). β-actin was used as an internal reference control. Immunofluorescence staining (**M**) and E-selectin positive area (**N**) were used to assess activation of E-selectin(× 630, Scale bar = 10 μm, *n* = 6 per group). Results are expressed as mean ± SEM; ^***^P < 0.001 vs. Cxcr2^L/L^group; ^##^*P* < 0.01, and ^###^*P* < 0.001 vs. DKD-Cxcr2^L/L^ group

To further explore the role of CXCR2 in glycocalyx dysfunction in GECs under DKD conditions, glycocalyx degradation fragments (syndecan-1 and heparan sulfate) were detected. The immunofluorescence results revealed a remarkable reversal of decreased levels of heparan sulfate (Fig. 3A, B) and syndecan-1 (Fig. 3D, E) in the glomeruli of DKD-C*xcr*2^L/L^ mice following endothelial *Cxcr2* knockout. Intriguingly, the serum level of heparan sulfate (Fig. 3C) and syndecan-1 (Fig. 3F) was dramatically increased in DKD-*Cxcr2* ^L/L^ group compared to the DKD-*Cxcr2* ^eCKO^ group. Surprisingly, endothelial Cxcr2 knockout resulted in a significant increase in glycocalyx depth and glycocalyx coverage compared to the DKD-Cxcr2^L/L^ group (Fig. 3G-I), consistent with the results of the immunofluorescence. Cxcr2 knockout also ameliorated podocyte foot process fusion (Fig. 3J). Due to a vicious cycle between inflammation and glycocalyx degradation, we explored whether CXCR2 also regulated chronic inflammation. As shown in Fig. S3, the kidneys from DKD-*Cxcr2* ^eCKO^ mice exhibited decreased infiltration of inflammatory cells (including neutrophils and macrophages) compared with DKD-*Cxcr2* ^L/L^ mice (Fig. S3A-D). Cxcr2 mRNA level was significantly higher in the DKD group (Fig. S3E). Moreover, the mRNA levels of chemokines in the glomeruli (*Mcp-1 (Ccl2), Ccl5, Cxcl1, Cxcl2*(Fig. S3F) and the serum expression of (TNF-α, IL-1β, IL-6, and IL-18)(Fig. S3G-J) were lower in DKD-*Cxcr2* ^eCKO^ group compared with DKD-*Cxcr2* ^L/L^ group. We also found that the phosphorylation levels of p-IKKβ, p-IκBα, and p-NF-κBp65 in the glomeruli of DKD-*Cxcr2* ^eCKO^ mice were significantly decreased compared with DKD-*Cxcr2* ^L/L^ mice (Fig. 3K, L). E-selectin, involved in the induction of endothelial injury as the NF-κB target gene in ECs, exhibited a notable increase in expression in the DKD-Cxcr2^L/L^groups. However, it was significantly inhibited by the endothelial cell-specific knockout of Cxcr2 (Fig. 3M, N).

### CXCR2 silencing attenuate high glucose-induced inflammation and glycocalyx shedding in GECs by NF-κB p65 signaling

**Fig. 4:**
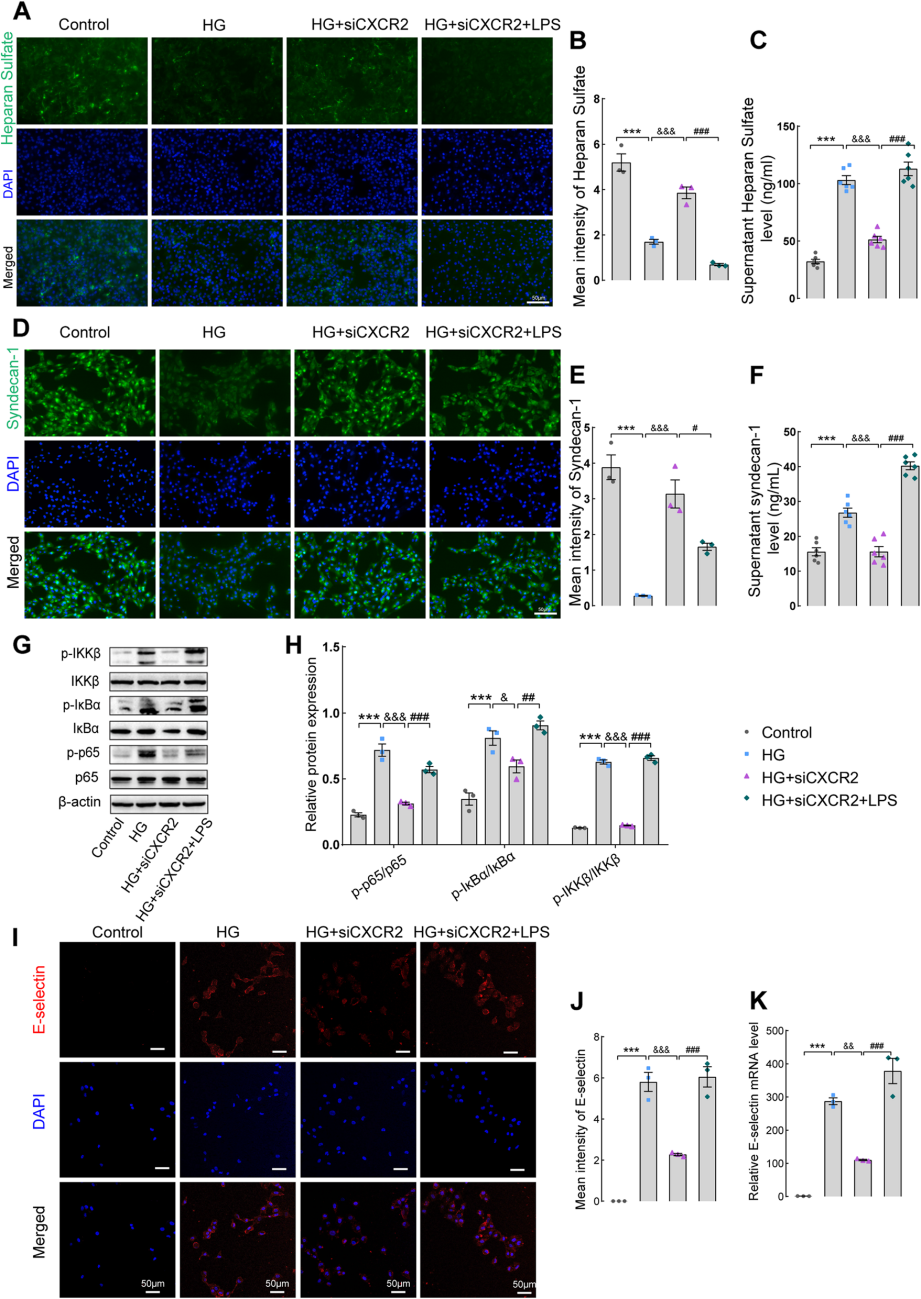
CXCR2 knockout in GECs ameliorates renal glycocalyx degradation by the NF-κB p65 signaling pathway. **A** Immunofluorescence was used to detect the expression of heparan sulfate in GECs. **B** Mean intensity of heparan sulfate (× 200, Scale bar = 50 μm, *n* = 3). **C** ELISA was used to measure the level of heparan sulfate in the supernatant of four groups of cells.** D** Immunofluorescence was used to detect the expression of syndecan-1 in GECs. **E** Mean intensity of syndecan-1(× 200, Scale bar = 50 μm, *n* = 3). **F** ELISA was used to measure the levels of syndecan-1 in the supernatant. **G** and **H** The protein levels of p-IKKβ, IKKβ, p-IκBα, IκBα, p-NF-κBp65, and NF-κB p65 were detected by western blotting. β-Actin was used as an internal reference control. The immunofluorescence staining (**I**) and mean intensity of E-selectin (**J**) (× 200, Scale bar = 50 μm, *n* = 3). qPCR assay was used to test the level of E-selectin (**K**). Representative images were shown; original magnification× 200. Results are expressed as mean ± SEM; ***P < 0.001 vs. control group; ^&^ *P* < 0.05, ^&&^ *P* < 0.01,^&&&^ *P* < 0.001 vs. HG group; ^#^ *P* < 0.05, ^##^ *P* < 0.01, ^###^ *P* < 0.001vs. HG + SiCXCR2 group; HG, high glucose

To further investigate whether CXCR2 regulates glycocalyx shedding by the NF-κB p65 signaling pathway, GECs were treated with high glucose and LPS (an activator of NF-κB signaling). The mRNA and protein expression of CXCR2 transfected with CXCR2 siRNA1, siRNA2, and siRNA3 in GECs were shown in Fig. S4A-C, and siRNA 3 interference was selected for subsequent experiments. Moreover, as shown in Fig. S5A, the CXCR2 mRNA level was detected firstly. As anticipated, silencing CXCR2 in GECs led to increased expression of heparan sulfate and syndecan-1 under high-glucose microenvironment. Importantly, the addition of LPS resulted in a decrease in heparan sulfate and syndecan-1 expression in GECs and an increase in supernatant compared to the HG + SiCXCR2 group (Fig. 4A-F). Additionally, in the high-glucose microenvironment, LPS was added into the CXCR2 siRNA group (HG + SiCXCR2 + LPS group), the decreased levels of p-IKKβ, p-IκBα, and p-NF-κBp65 in the CXCR2 siRNA group were significantly upregulated again.(Fig. 4G, H). The downregulated mRNA levels of inflammatory cytokines (TNF-α, IL-1β, IL-6, and MCP-1) in the HG + SiCXCR2 group were remarkably upregulated again after activating NF-κB signaling by adding LPS (Fig. S5B). Interfering with CXCR2 expression in GECs also led to a reduction in CXCL1 (Fig. S5C) and CXCL8 (Fig. S5D) in the cell supernatant, and these levels were upregulated again by adding LPS. In Fig. 4I-K, we found that endothelial cell activation induced by high glucose (HG) was significantly reduced following CXCR2 siRNA treatment, and this inhibitory effect was reversed by LPS. Surprisingly, although adding LPS reversed the inflammation suppression and glycocalyx shedding improvement brought about by silencing CXCR2. However, compared with the HG + LPS + siCXCR2 group, the inflammatory factors activation and glycocalyx shedding were more intense in the HG + LPS group, and silencing CXCR2 could still play a certain improvement role, both in the levels of glycocalyx sheeding (Fig. S6A-B), levels of inflammatory factors (Fig. S6D), and NF-κB p65 signaling pathway (Fig. S6E-F). The above data indicate that targeted inhibition of CXCR2 play a critical anti-inflammatory role in GECs by NF-κB p65 signaling.

### CXCR2 overexpression exacerbates inflammation and glycocalyx shedding by activating the NF-κB p65 signaling pathway in GECs

**Fig. 5:**
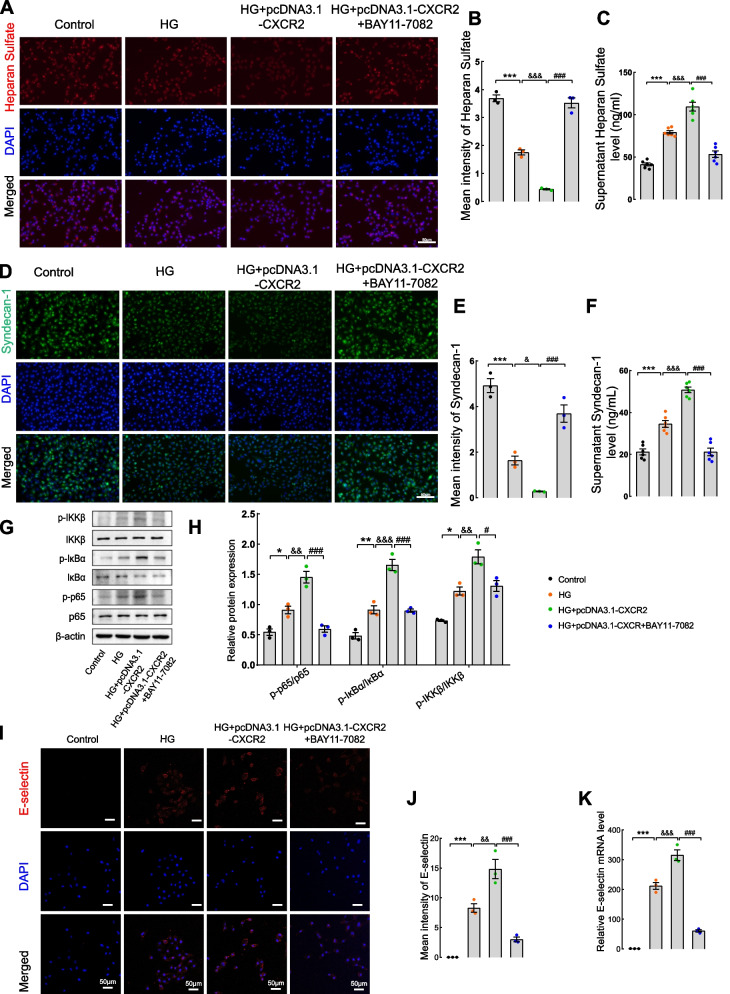
CXCR2 overexpression in GECs aggravates renal glycocalyx degradation and inflammation via the NF-κB p65 signaling pathway. **A** Immunofluorescence was used to detect the expression of heparan sulfate in GECs. **B** Mean intensity of heparan sulfate (× 200, Scale bar = 50 μm, *n* = 3). **C** ELISA was used to measure the level of heparan sulfate in the supernatant of four groups of cells**. D** Immunofluorescence was used to detect the expression of syndecan-1 in GECs. **E** Mean intensity of syndecan-1 (× 200, Scale bar = 50 μm, *n* = 3). **F** ELISA was used to measure the level of syndecan-1 in the supernatant. **G** and **H** The protein levels of p-IKKβ, IKKβ, p-IκBα, IκBα, p-NF-κB p65, and NF-κB p65 were detected by western blotting. β-Actin was used as an internal reference control. The immunofluorescence staining (**I**) and mean intensity of E-selectin (**J**) (× 200, Scale bar = 50 μm, *n* = 3). qPCR assay was used to test the level of E-selectin (**K**). Representative images were shown. Results are expressed as mean ± SEM; **P < 0.01 vs. control group; &&*P* < 0.01 vs. HG + pcDNA3.1 group; ##*P* < 0.01 vs. HG + pcDNA3.1-CXCR2group; ^*^ *P* < 0.05, ^**^ *P* < 0.01 and ^***^ *P* < 0.001 vs. control group; ^&^ *P* < 0.05, ^&&^ *P* < 0.01, ^&&&^ *P* < 0.001 vs. HG group; ^#^ *P* < 0.05, ^###^ *P* < 0.001 vs. HG + pcDNA3.1-CXCR2 group; HG:high glucose

To further validate the molecular mechanism of CXCR2 in inflammation and glycocalyx shedding upon overexpressing CXCR2 in GECs, the NF-κB inhibitor BAY11–7082 was introduced into the culture medium. The mRNA and protein expression of CXCR2, transfected with pcDNA3.1-CXCR2 in GECs,was illustrated in Fig. S4D-F. In Fig. S7A, the CXCR2 mRNA level in four group was detected. Similarly, immunofluorescence experiments showed that, compared to the HG group, heparan sulfate and syndecan-1 exhibited a more significant decrease following CXCR2 overexpression. Intriguingly, the immunofluorescence intensity were increased after inhibiting NF-κB signaling. The increased levels of heparan sulfate and syndecan-1 in the supernatant of the CXCR2 overexpression group were reversed by BAY11–7082 (Fig. 5A-F). Crucially, the phosphorylation of IKKβ, IκBα, and NF-κB p65 was significantly increased in the CXCR2 overexpression group, which was markedly ameliorated by BAY11–7082 (Fig. 5G-H). Likewise, significant increase of *TNF-α, IL-1β, IL-6, MCP-1* (Fig. S7B) and *CXCL1,CXCL8* mRNA levels (Fig. S7C-D) were abolished upon the addition of BAY11–7082. In Fig. 5I-K, endothelial cell activation was higher after overexpression of CXCR2 compared to the HG group. Additionally, the expression of E-selectin induced by CXCR2 overexpression was reversed by BAY11–7082. In summary, the aforementioned studies suggest that CXCR2 knockout could ameliorate glycocalyx shedding and endothelial cell activation induced by inflammatory factors by reducing NF-KB pathway activation.

### CXCR2 modulates the expression of glycocalyx shedding enzymes via the NF-κB pathway

**Fig. 6:**
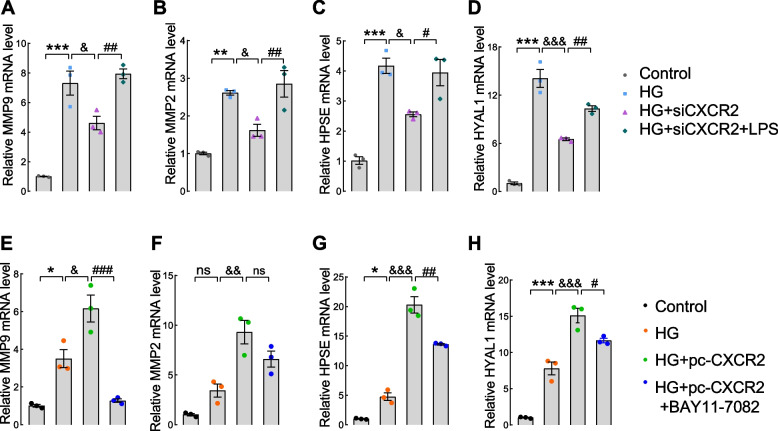
The expression of glycocalyx shedding enzymes qPCR assay was used to test the mRNA levels of MMP9 (**A**), MMP2 (**B**),HPSE (**C**) and HYAL1 (**D**) in the Control, HG, HG + siCXCR2 and HG + siCXCR2 + LPS group. The mRNA levels of MMP9 (**E**), MMP2 (**F**), HPSE (**G**) and HYAL1 (**H**) in Control, HG, HG + pcDNA3.1-CXCR2 and HG + pcDNA3.1-CXCR2 + BAY11–7082 group

To delve into the potential involvement of CXCR2 in hydrolase generation via the NF-κB pathway, we assessed the mRNA levels of matrix metalloproteinases (MMPs), hyaluronidase-1 (HYAL1), and heparanase (HPSE) in GECs transfected with siCXCR2 (Fig. 6A-D) and pcDNA3.1-CXCR2 (Fig. 6E-H). The results showed an increase in shedding enzymes mRNA level in the HG group, which correspondingly decreased or increased with silence or overexpression of CXCR2. Notably, CXCR2 modulates the NF-κB pathway to regulate the production of shedding enzymes from the transcriptional level.

## Discussion

Indeed, chronic inflammatory response has been recognized as a crucial contributor to the pathogenesis of DKD [41, 42]. Elucidating the mechanisms underlying the occurrence of chronic inflammation in the kidneys holds paramount importance in decelerating DKD progression. Previous studies have provided evidence that chemokines and their receptors have a significant impact on the exacerbation of inflammation related to diabetes, potentially accelerating the deterioration of organ damage caused by high glucose, particularly in the eyes and kidneys [43–46].

CXCR2 is a receptor involved in mediating the effects of certain chemokines, serves as a signaling molecule regulating and plays a crucial role in inflammation and tissue damage [47]. The research findings has demonstrated that elevated expression of CXCR2 in the glomeruli of patients with DKD, a pattern supported by data in the database indicating that CXCR2 levels have significant diagnostic value for DKD. Consistent with our study is the fact that previous studies have provided evidence of the involvement of CXCR2 in various cellular processes and pathways related to renal pathology in DKD [48, 49]. E-selectin, a marker of endothelial activation, is typically expressed by endothelial cells (ECs) in rapid response to inflammatory stimuli [50, 51]. Our study suggests that the glomerular endothelium is in an activated state accompanied by glycocalyx shedding. This process is associated with a decline in the filtration barrier function of the glomeruli. CXCR2 is likely involved in the inflammatory process within the glomerular endothelium, promoting the occurrence and progression of DKD. However, more research is needed to elucidate the specific functions and underlying mechanisms by which CXCR2 contributes to glomerular endothelial inflammation in DKD.

To investigate the involvement of endothelial CXCR2 in DKD development, conditional knockout mice targeting *Cxcr2* in glomerular endothelium were constructed (*Cxcr2* ^L/L,^ *Cxcr2* ^eCKO^), The research results have demonstrated that knocking out CXCR2 in GECs could improve renal function in DKD mice. This suggests that CXCR2 in endothelial cells plays a significant role in promoting the occurrence and progression of DKD. This effect may be associated with changes in endothelial structure and alterations in the inflammatory environment.

During the early stages of kidney disease, one of the most characteristic alterations is the dysfunction of the glomerular endothelium and the loss of the glycocalyx, a layer of protective molecules covering the endothelial cells lining the blood vessels, including those in the kidneys [52, 53]. The disruption or shedding of the glycocalyx has been associated with numerous inflammatory conditions, including DKD [21, 54]. Our research also found that inflammation in the kidneys of diabetic nephropathy mice is exacerbated, and there is severe shedding of the glycocalyx in the glomerular endothelium. However, after knocking out CXCR2 in the endothelium, both inflammation and glycocalyx shedding are significantly reduced. This indicates that inhibiting endothelial CXCR2 expression improves the glomerular filtration barrier, reduces proteinuria by decreasing inflammation and glycocalyx shedding.

In the context of DKD, both endothelial dysfunction and inflammation are recognized as contributors to disease progression [55–58]. However, the specific relationship between endothelial inflammation and glycocalyx shedding in DKD remains unclear. Furthermore, the nuclear factor-κB (NF-κB) signaling pathway is known to play a pivotal role in high glucose-induced renal inflammation in GECs [59–61]. In addition to common cytokines such as TNF-α, IL-1β, IL-6, and IL-18, which are known to accelerate inflammatory responses in the endothelium [23, 24], recent studies have underscored the importance of key chemokines such as MCP-1, CCL5, CXCL1, and CXCL2 in promoting inflammation in DKD by facilitating the recruitment of immune cells into the glomeruli [62–64]. Our results demonstrate that CXCR2 might mediate an inflammatory response by the NF-κB p65 signaling pathway to bridge glycoclyx dysfunction，and *Cxcr2* knockout in endothelial cells might disrupt the vicious circle between inflammation and glycocalyx shedding by the NF-κB p65 signaling pathway.

Many studies have indicated that the shedding of endothelial glycocalyx is associated with specific enzymes [21, 27, 65], such as matrix metalloproteinases (MMPs) which lead to glycocalyx shedding by cleaving the core protein like syndecans. Other enzymes, such as HYAL and HPSE, are capable of hydrolyzing Hyaluronic and heparan sulfate. In the study by Raina et al. [21], it was shown that matrix metalloproteinase (MMP)-2 and MMP-9 play a role in mediating glycocalyx damage in GECs, subsequently increasing the permeability of urinary proteins. Preserving the integrity of the glycocalyx could reduce the production of proteinuria and prevent the deterioration of kidney function in DKD. Our evidence above suggests that CXCR2 knockout reduces endothelial cell activation and inflammatory factor release by inhibiting the NF-κB pathway, thereby mitigating shedding enzymes and protecting the glycocalyx.

**Fig. 7:**
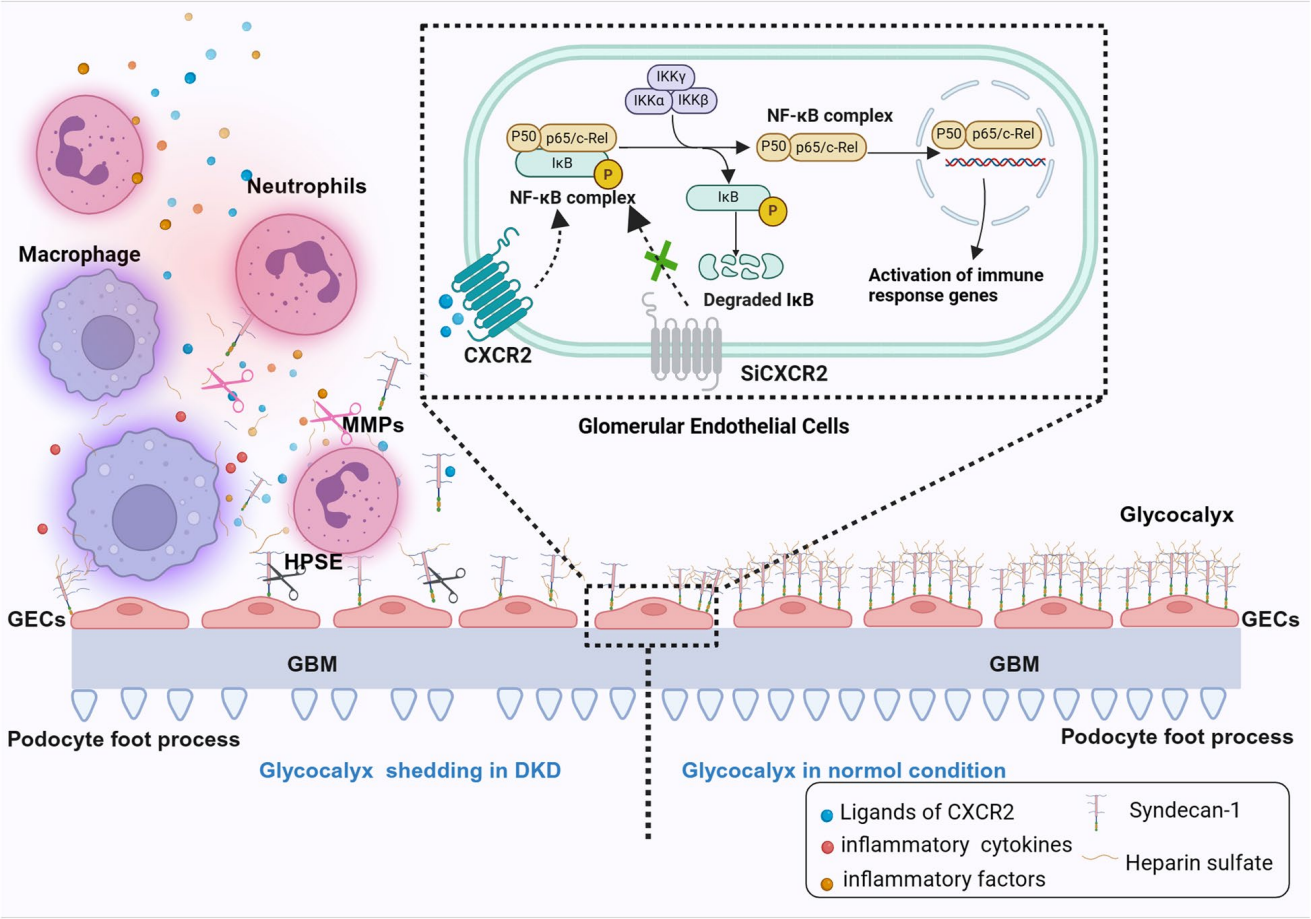
Schematic of the mechanism by which CXCR2 bridge the vicious circle between inflammation and glycocalyx degradation by NF-κB p65 signaling pathway. Graphical representation depicting how endothelial CXCR2 deficiency attenuates renal inflammation and glycocalyx shedding through NF-κB signaling in diabetic kidney disease. GEnC and endothelial glycocalyx, GBM and podocytes form the glomerular filtration barrier. CXCL1 and CXCL8, as ligands of CXCR2, bind to CXCR2 and initiate the NF-κB p65 signaling pathway. In DKD, this activation leads to the release of inflammatory factors, endothelial cell activation, and recruitment of macrophages and neutrophils to the endothelial cell surface. These recruited cells further release inflammatory factors and chemokines, ultimately resulting in glycocalyx shedding. Shedding of the endothelial glycocalyx triggers glomerular inflammation and may also initiate crosstalk between endothelial cells and podocytes, leading podocyte injury (podocyte foot processes loss) and leading to proteinuria. Endothelial CXCR2 knockout significantly improves this process

Our findings suggest that in the early stages of chronic renal injury in the diabetic microenvironment, CXCR2 activation by its ligands initiates the upregulation of various proinflammatory factors and chemokines through the NF-κB signaling pathway. This process leads to the recruitment and adhesion of immune cells, including macrophages, neutrophils, and other immune infiltrating cells, at the site of glomerular injury, releasing inflammatory mediators. More importantly, inflammation-induced stimulation compromises glycocalyx integrity in GECs, leading to glycocalyx shedding. This degradation results in the formation of syndecan-1 and heparan sulfate fragments, further fueling inflammation, and creating a detrimental feedback loop between inflammation and glycocalyx shedding. Notably, the specific knockout of CXCR2 in the endothelium attenuates glycocalyx shedding in the glomeruli by inhibiting the overactivation of the immune inflammatory response (Fig. 7).

## Conclusions

Our findings highlight the significance of CXCR2 and its pivotal role in the pathogenesis of DKD. Specifically, CXCR2 plays a crucial role in NF-κB activation, inflammation regulation, and subsequent glycocalyx shedding in GECs. Targeting CXCR2 has the potential for attenuating inflammation and protect against endothelial glycocalyx damage in the kidneys, potentially slowing DKD progression. Furthermore, employing tissue-specific targeting to inhibit CXCR2 suggests a potentially therapeutic strategy for DKD. However, it is imperative to acknowledge further research and clinical studies are necessary to validate these findings and explore the feasibility and efficacy of CXCR2 inhibition as a therapeutic avenue for DKD.

### Supplementary Information

**Additional file 1: Supplementary Fig. 1.** ligands of CXCR2 are increased and positively correlated with UACR. The serum levels of CXCL1 (A), CXCL8 (C), syndecan-1 (E),and syndecan-4 (G) were determined by human ELISA kit(*n* = 40 per group). And Line regression was used to assess the correlation between serum CXCL1 (B), CXCL8 (D) and syndecan-1 (F) expression with UACR in DKD (n = 40). Immunohistochemical staining (H) and scores (I) for the expression of CXCR2 are shown in glomeruli and enlarged glomerular image of the box (× 400, Scale bar = 50 μm, *n* = 3 for each group).UACR, urine albumin creatine ratio; DKD, diabetic kidney disease; Error bars indicate SEM, and data represent mean ± SEM. **P < 0.01, ***P < 0.001 vs. control group.

**Additional file 2: Supplementary Fig. 2.** Endothelial CXCR2 knockout efficiency and effect on CXCR2 knockout mice. (A) Schematic representation of recombination between loxP sites and CXCR2 alleles. loxP sites are indicated as yellow triangles, and PCR primer positions are indicated by arrows. (B) Agarose gel electropherograms showing the results of PCR amplification using primers from genomic DNA isolated from tail tissue of wild-type and *Cxcr2*^eCKO^ mice. *Cxcr2*^eCKO^ mice showed both the primer1 fragment (208 bp) and primer2 (179 bp), and the wild-type mice showed a 135 bp fragment of primer1. (C) Immunofluorescence staining of CD31 was used to identify mouse glomerular endothelial cells (MGECs) (× 200, Scale bar = 50 μm, *n* = 3). Relative *Cxcr2* mRNA level (D) and relative protein expression (E and F) of isolated MGECs from *Cxcr2*^L/L^ and *Cxcr2*^eCKO^ mice were tested. Blood glucose and body weight were monitored biweekly and were shown at the ages of 16 weeks and 28 weeks (G and H). (I) Plasma cholesterol (TC) and (J) plasma triglycerides (TG) were measured in four groups. Results are expressed as mean ± SEM (*n* = 6); **P < 0.01, and ***P < 0.001 vs. CXCR2 ^L/L^ group; ^ns^*P* > 0.05.

**Additional file 3: Supplementary Fig. 3.** Renal inflammatory response in four groups of mice. Immunohistochemistry was used to evaluate the infiltration of macrophages (F4/80) (A) and neutrophils (MPO) in the kidneys of mice (B). Positive area of F4/80 (C) and MPO (D) (× 400, Scale bar = 50 μm, *n* = 3) were tested. And qPCR experiments was used to quantify *cxcr2* mRNA expression in four groups of mice(E). (F) The mRNA levels of *(MCP-1 (Ccl2), Ccl5, Cxcl1 and Cxcl2* in the glomeruli of mice were detected (*n* = 3). ELISA was used to measure the levels of TNF-α (G), IL-1β (H), IL-6 (I), and IL-18 (J) in the peripheral blood serum of the four groups of mice (*n* = 8). ImageJ was used for quantitative analysis of the positive staining area. Representative images were shown; Results are expressed as mean ± SEM; ^**^P < 0.01, ^***^P < 0.001vs. CXCR2^L/L^ group; ^#^*P* < 0.05, ^##^*P* < 0.01, ^###^*P* < 0.001 vs. DKD-CXCR2^L/L^ group. ^&&&^*P* < 0.001, CXCR2^eCKO^ group vs. CXCR2^L/L^ group.

**Additional file 4: Supplementary Fig. 4.** CXCR2 silence and overexpression efficiency in GECs*.* The mRNA(A) and protein(B and C) level of CXCR2 after transfection with CXCR2 siRNA1, siRNA2, siRNA3 in GECs, universal negative control siRNA was used as a control. The mRNA(D) and protein(E and F) level of CXCR2 transfection with pcDNA3.1-CXCR2 in GECs, the universal negative control siRNA or the pcDNA3.1 empty vector was used as a control. Results are expressed as mean ± SEM; ***P < 0.001 vs. control group; ^ns^*P* > 0.05.

**Additional file 5: Supplementary Fig. 5.** The inflammation in CXCR2 knockout GECs. The qPCR assay employed to quantify the level of CXCR2 in four groups. In the two siCXCR2 group, the expression were minimal, demonstrating that CXCR2 was successfully silenced (A). *TNF-α, IL-1β, IL-6, and MCP-1* levels in four groups of GECs were also tested by qPCR (B). Elisa was used to test the levels of CXCL1(C) and CXCL8(D) in supernatant. Representative images were shown; Results are expressed as mean ± SEM;*P < 0.05, **P < 0.01, ***P < 0.001 vs. control group; ^&&^*P* < 0.01,^&&&^*P* < 0.001 vs. HG group; ^##^*P* < 0.01, ^###^*P* < 0.001 vs. HG + SiCXCR2 group; HG, high glucose.

**Additional file 6: Supplementary Fig. 6.** The levels of inflammatory factors and glycocalyx shedding in HG + LPS group and HG + LPS + siCXCR2 group. ELISA was used to measure the levels of heparan sulfate (A) and syndecan-1(B) in the supernatant in GECs of two groups. (C) The CXCR2 mRNA level in two groups. (D) qPCR experiments was used to quantify inflammatory factors mRNA levels. (E and F) The protein levels of syndecan-1, p-IKKβ, IKKβ, p-IκBα, IκBα, p-NF-κBp65, and NF-κB p65 were detected by western blotting. β-Actin was used as an internal reference control (*n* = 3). Results are expressed as mean ± SEM; *P < 0.05,**P < 0.01,***P < 0.001, HG + siCXCR2 + LPS vs. HG + LPS group; the universal negative control siRNA was used as a control.

**Additional file 7: Supplementary Fig. 7.** The inflammation in CXCR2 overexpression GECs. (A) The mRNA level of CXCR2 in four groups was detected. And we also tested the TNF-α, IL-1β, IL-6, and MCP-1 mRNA levels .(n = 3)(B). *CXCL1*(C) and *CXCL8* (D) mRNA level in GECs of four groups. Results are expressed as mean ± SEM; *P < 0.05, **P < 0.01, ***P < 0.001 vs. control group; ^&&&^*P* < 0.01 vs. HG group; ^###^*P* < 0.001 vs. HG + pcDNA3.1-CXCR2group; HG, high glucose; ^ns^*P* > 0.05.

**Additional file 8: Supplementary Table 1.** The plasma and urine variables from different groups of humans. DKD, diabetic kidney disease; BMI, body mass index; FPG, fasting plasma glucose; HbA1c, glycosylated hemoglobin; BUN, blood urea nitrogen; Scr, Serum creatinine; eGFR, estimated glomerular filtration rate; UACR, urine albumin creatinine ratio; α1-MG, α1-microglobulin; RBP, retinol-binding protein; Compared with the Control group, ^**^P<0.01.

**Additional file 9: Supplementary Table 2.** The CXCR2 siRNA sequences.

**Additional file 10: Supplementary Table 3.** Primer sequences of *Mus musculus* used for qRT-PCR.

**Additional file 11: Supplementary Table 4.** Primer sequences of *Homo sapiens* used for qRT-PCR

## Data Availability

All data needed to evaluate the conclusions in the paper are present in the paper and/or the Supplementary Materials.

## Abbreviations

BMI: Body mass index
BUN: Blood urea nitrogen
CXCR2: C-X-C motif chemokine receptor 2
CXCL1: C-X-C motif chemokine ligand 1
CXCL8: C-X-C motif chemokine ligand 8
Ccl5: C-C motif chemokine ligand 5
Cxcl2: C-X-C motif chemokine ligand 2
DKD: Diabetic kidney disease
eGFR: Estimated glomerular filtration rate
ECL: Enhanced chemiluminescent
FPG: Fasting plasma glucose
FBS: Fetal bovine serum
HRP: Horseradish peroxidase
HFD: High-fat diet
HbA1c: Glycosylated hemoglobin
IL-1β: Interleukin 1 beta
IL-6: Interleukin 6
IL-18: Interleukin 18
LPS: Lipopolysaccharides
MCP-1 (Ccl2): C-C motif chemokine ligand 2
PBS: Phosphate-buffered saline
RBP: Retinol-binding protein
Scr: Serum creatinine
TNF-α: Tumor necrosis factor
TC: Total cholestero
TG: Triglyceride
UACR: Urine albumin creatinine ratio
α1-MG: α1-microglobulin
