# Supplementary material for: Endothelial CXCR2 deficiency attenuates renal inflammation and glycocalyx shedding through NF-κB signaling in diabetic kidney disease
Source: Cell Commun Signal. 2024 Mar 25;22:191. doi: 10.1186/s12964-024-01565-2 (PMC10964613; doi:10.1186/s12964-024-01565-2)

**Supplementary Fig 1 . ligands of CXCR2 are increased and positively correlated with UACR**.

The serum levels of CXCL1**(A)**, CXCL8**(C)**, syndecan-1**(E)**,and syndecan-4**(G)** were determined by human ELISA kit(n=40 per group). And Line regression was used to assess the correlation between serum CXCL1**(B)**, CXCL8**(D)** and syndecan-1**(F)** expression with UACR in DKD(n=40). Immunohistochemical staining **(H)** and scores **(I)** for the expression of CXCR2 are shown in glomeruli and enlarged glomerular image of the box (x400, Scale bar = 50μm, n = 3 for each group).UACR, urine albumin creatine ratio; DKD, diabetic kidney disease; Error bars indicate SEM, and data represent mean ± SEM. **P < 0.01, ***P < 0.001 vs. control group.


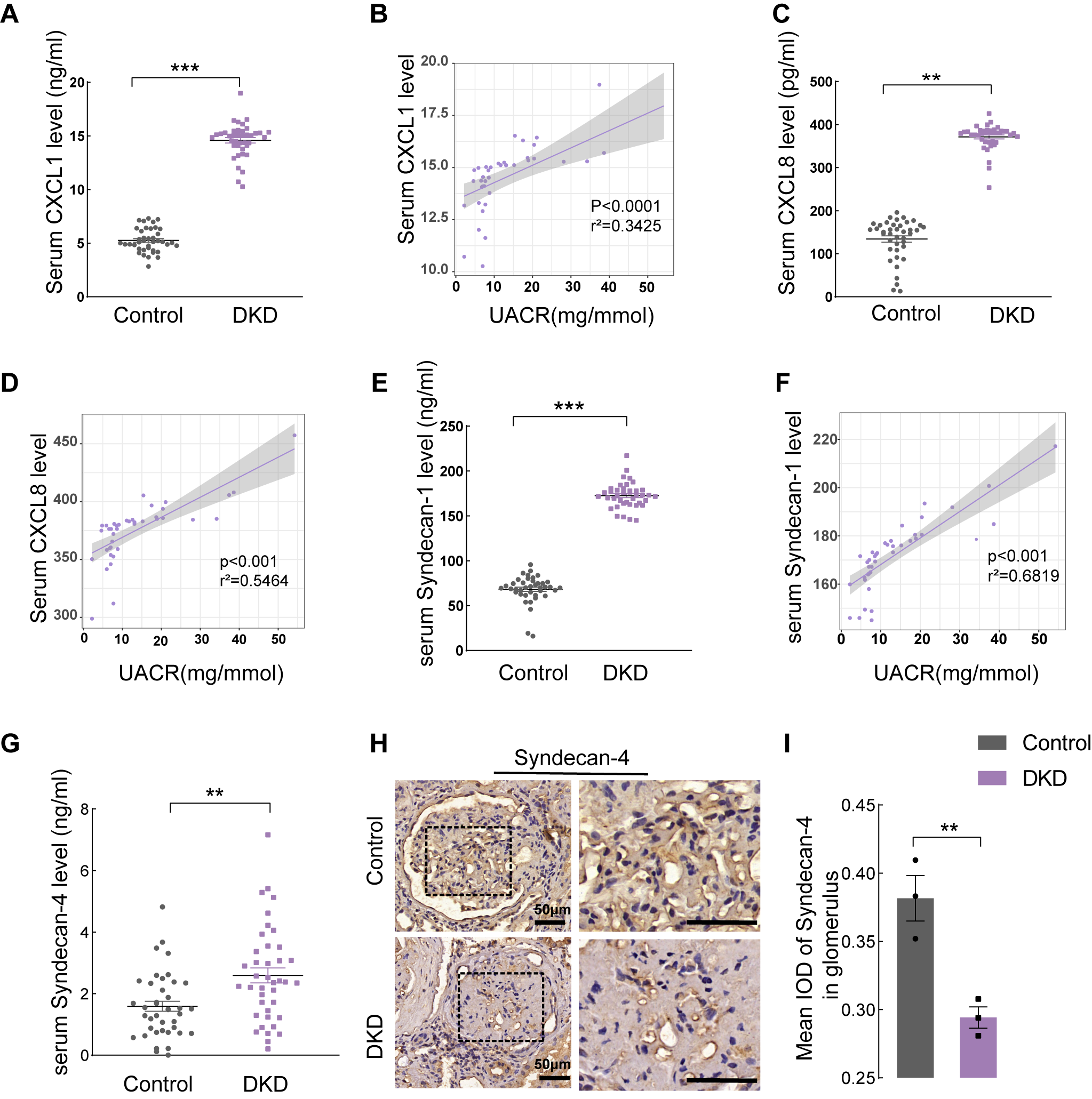

Supplement: Supplementary file 1 — Additional file 1: Supplementary Fig. 1. ligands of CXCR2 are increased and positively correlated with UACR. The serum levels of CXCL1 (A), CXCL8 (C), syndecan-1 (E),and syndecan-4 (G) were determined by human ELISA kit(n = 40 per group). And Line regression was used to assess the correlation between serum CXCL1 (B), CXCL8 (D) and syndecan-1 (F) expression with UACR in DKD (n = 40). Immunohistochemical staining (H) and scores (I) for the expression of CXCR2 are shown in glomeruli and enlarged glomerular image of the box (× 400, Scale bar = 50 μm, n = 3 for each group).UACR, urine albumin creatine ratio; DKD, diabetic kidney disease; Error bars indicate SEM, and data represent mean ± SEM. **P < 0.01, ***P < 0.001 vs. control group. [file 12964_2024_1565_MOESM1_ESM.docx]
