# Supplementary material for: Endothelial CXCR2 deficiency attenuates renal inflammation and glycocalyx shedding through NF-κB signaling in diabetic kidney disease
Source: Cell Commun Signal. 2024 Mar 25;22:191. doi: 10.1186/s12964-024-01565-2 (PMC10964613; doi:10.1186/s12964-024-01565-2)

**Supplementary Fig 2. Endothelial CXCR2 knockout efficiency and effect on CXCR2 knockout mice. (A)** Schematic representation of recombination between loxP sites and CXCR2 alleles. loxP sites are indicated as yellow triangles, and PCR primer positions are indicated by arrows. **(B)** Agarose gel electropherograms showing the results of PCR amplification using primers from genomic DNA isolated from tail tissue of wild-type and *Cxcr2*^eCKO^ mice. *Cxcr2*^eCKO^ mice showed both the primer1 fragment (208 bp) and primer2 (179 bp), and the wild-type mice showed a 135 bp fragment of primer1. **(C)**Immunofluorescence staining of CD31 was used to identify mouse glomerular endothelial cells (MGECs) **(**×200, Scale bar=50μm, n=3**)** . Relative *Cxcr2* mRNA level **(D)** and relative protein expression **(E and F)** of isolated MGECs from *Cxcr2*^L/L^ and *Cxcr2*^eCKO^ mice were tested. Blood glucose and body weight were monitored biweekly and were shown at the ages of 16 weeks and 28 weeks **(G and H)**. **(I)** Plasma cholesterol (TC) and **(J)** plasma triglycerides (TG) were measured in four groups. Results are expressed as mean ± SEM (n = 6); **P< 0.01, and ***P< 0.001 vs. CXCR2 ^L/L^ group; ^ns^ P>0.05.


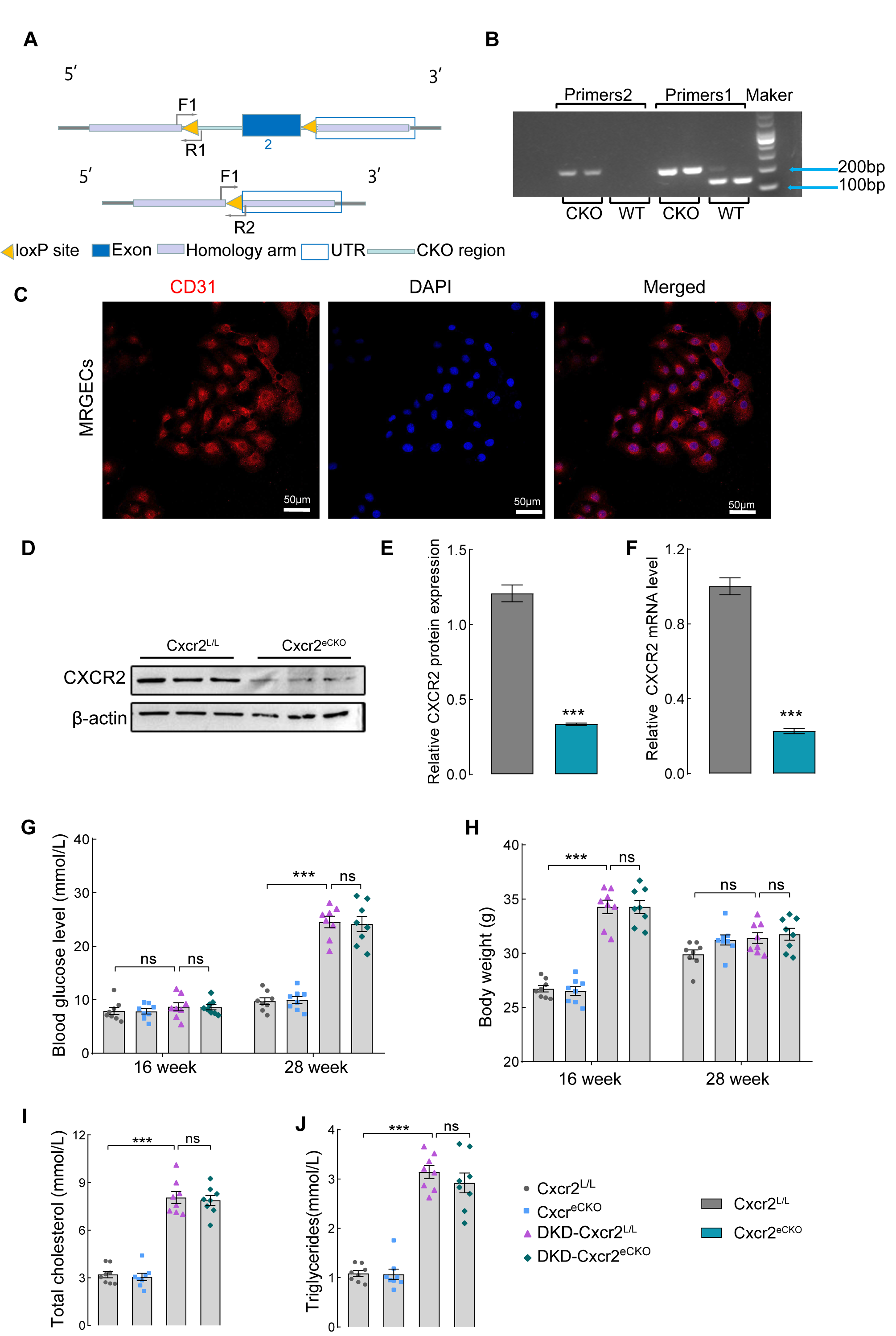

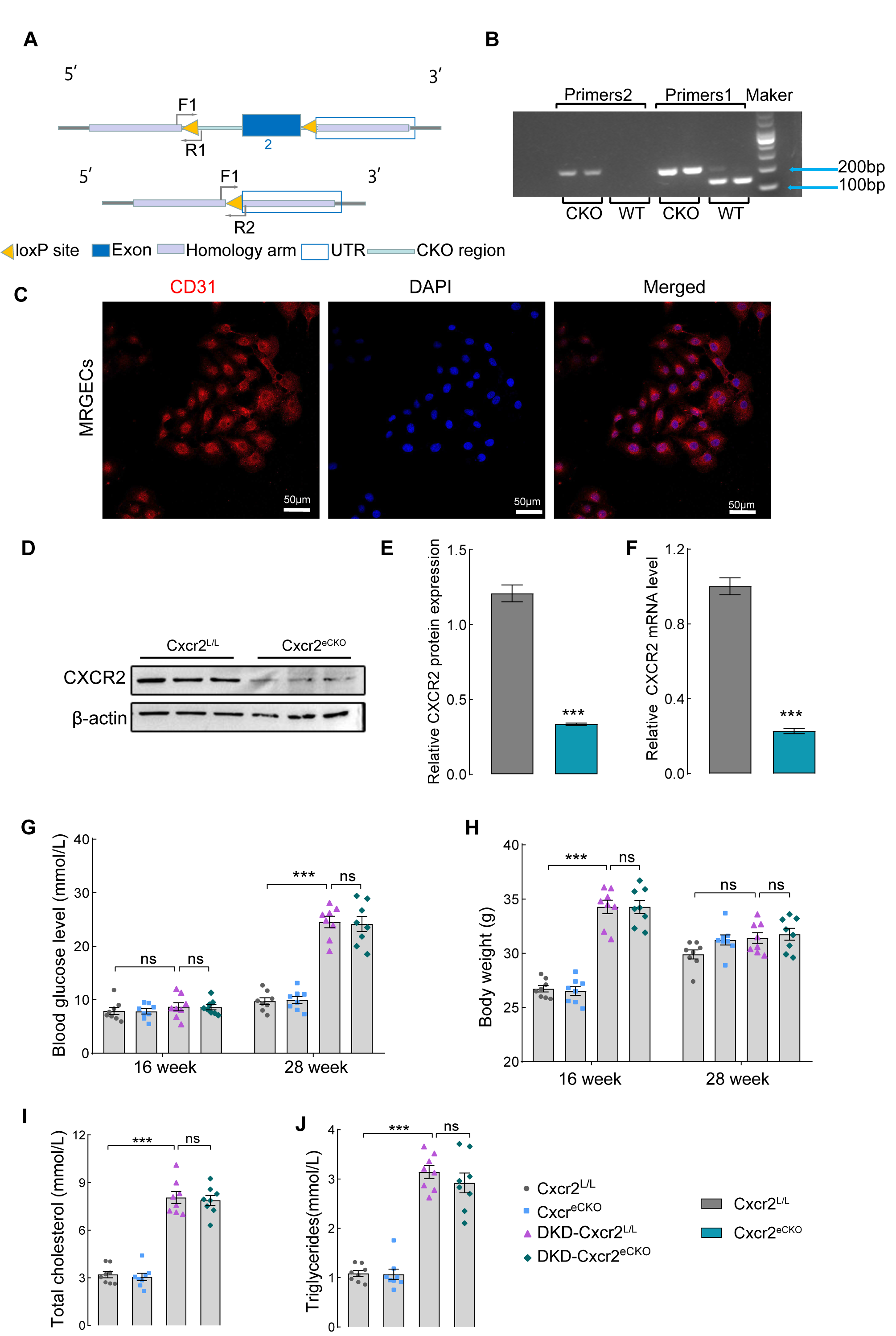

Supplement: Supplementary file 2 — Additional file 2: Supplementary Fig. 2. Endothelial CXCR2 knockout efficiency and effect on CXCR2 knockout mice. (A) Schematic representation of recombination between loxP sites and CXCR2 alleles. loxP sites are indicated as yellow triangles, and PCR primer positions are indicated by arrows. (B) Agarose gel electropherograms showing the results of PCR amplification using primers from genomic DNA isolated from tail tissue of wild-type and Cxcr2eCKO mice. Cxcr2eCKO mice showed both the primer1 fragment (208 bp) and primer2 (179 bp), and the wild-type mice showed a 135 bp fragment of primer1. (C) Immunofluorescence staining of CD31 was used to identify mouse glomerular endothelial cells (MGECs) (× 200, Scale bar = 50 μm, n = 3). Relative Cxcr2 mRNA level (D) and relative protein expression (E and F) of isolated MGECs from Cxcr2L/L and Cxcr2eCKO mice were tested. Blood glucose and body weight were monitored biweekly and were shown at the ages of 16 weeks and 28 weeks (G and H). (I) Plasma cholesterol (TC) and (J) plasma triglycerides (TG) were measured in four groups. Results are expressed as mean ± SEM (n = 6); **P < 0.01, and ***P < 0.001 vs. CXCR2 L/L group; nsP > 0.05. [file 12964_2024_1565_MOESM2_ESM.docx]
