# Supplementary material for: Endothelial CXCR2 deficiency attenuates renal inflammation and glycocalyx shedding through NF-κB signaling in diabetic kidney disease
Source: Cell Commun Signal. 2024 Mar 25;22:191. doi: 10.1186/s12964-024-01565-2 (PMC10964613; doi:10.1186/s12964-024-01565-2)

**Supplementary Fig 5. The inflammation in CXCR2 knockout GECs**. The qPCR assay employed to quantify the level of CXCR2 in four groups. In the two siCXCR2 group, the expression were minimal, demonstrating that CXCR2 was successfully silenced**(A)**. *TNF-α, IL-1β, IL-6, and MCP-1* levels in four groups of GECs were also tested by qPCR**(B)**. Elisa was used to test the levels of CXCL1**(C)** and CXCL8**(D)** in supernatant. Representative images were shown; Results are expressed as mean ± SEM;*P< 0.05, **P< 0.01, ***P< 0.001 vs. control group; ^&&^P< 0.01,^&&&^P< 0.001 vs. HG group; ^##^P < 0.01, ^###^P < 0.001 vs. HG+SiCXCR2 group; HG, high glucose.


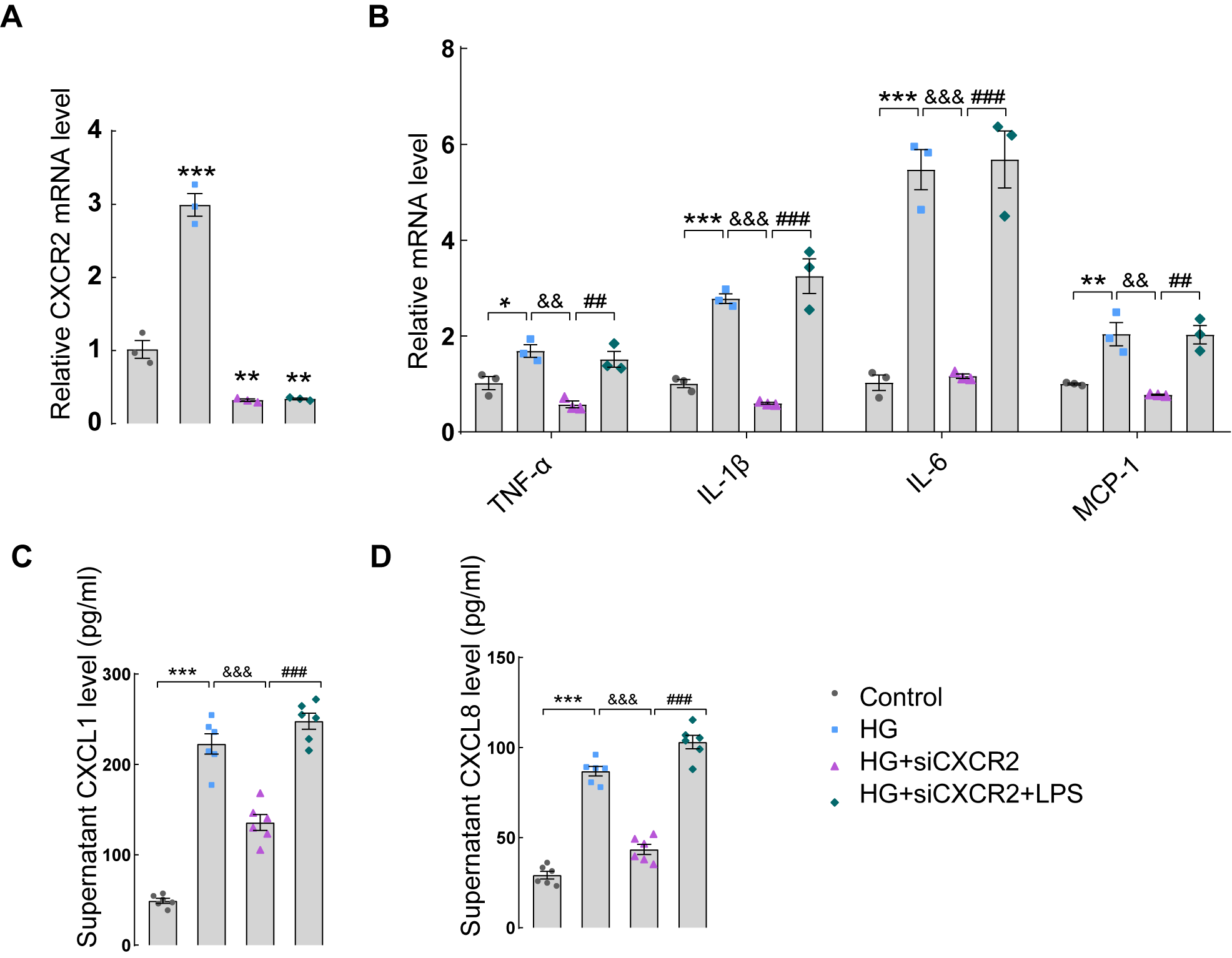

Supplement: Supplementary file 3 — Additional file 3: Supplementary Fig. 3. Renal inflammatory response in four groups of mice. Immunohistochemistry was used to evaluate the infiltration of macrophages (F4/80) (A) and neutrophils (MPO) in the kidneys of mice (B). Positive area of F4/80 (C) and MPO (D) (× 400, Scale bar = 50 μm, n = 3) were tested. And qPCR experiments was used to quantify cxcr2 mRNA expression in four groups of mice(E). (F) The mRNA levels of (MCP-1 (Ccl2), Ccl5, Cxcl1 and Cxcl2 in the glomeruli of mice were detected (n = 3). ELISA was used to measure the levels of TNF-α (G), IL-1β (H), IL-6 (I), and IL-18 (J) in the peripheral blood serum of the four groups of mice (n = 8). ImageJ was used for quantitative analysis of the positive staining area. Representative images were shown; Results are expressed as mean ± SEM; **P < 0.01, ***P < 0.001vs. CXCR2L/L group; #P < 0.05, ##P < 0.01, ###P < 0.001 vs. DKD-CXCR2L/L group. &&&P < 0.001, CXCR2eCKO group vs. CXCR2L/L group. [file 12964_2024_1565_MOESM3_ESM.docx]
