# Supplementary material for: Endothelial CXCR2 deficiency attenuates renal inflammation and glycocalyx shedding through NF-κB signaling in diabetic kidney disease
Source: Cell Commun Signal. 2024 Mar 25;22:191. doi: 10.1186/s12964-024-01565-2 (PMC10964613; doi:10.1186/s12964-024-01565-2)

**Supplementary Fig 6. The level of inflammatory factors and glycocalyx shedding in HG+LPS group and HG+LPS+siCXCR2 group.** ELISA was used to measure the level of heparan sulfate **(A)** and syndecan-1**(B)** in the supernatant in GECs of two groups. **(C)**The CXCR2 mRNA level in two groups. **(D)** qPCR experiments was used to quantify inflammatory factors mRNA level. **(E and F)** The protein levels of syndecan-1, p-IKKβ, IKKβ, p-IκBα, IκBα, p-NF-κBp65, and NF-κB p65 were detected by western blotting. β-Actin was used as an internal reference control (n=3). Results are expressed as mean ± SEM; *P< 0.05,**P< 0.01,***P< 0.001, HG+siCXCR2+LPS vs. HG+LPS group; the universal negative control siRNA was used as a control.


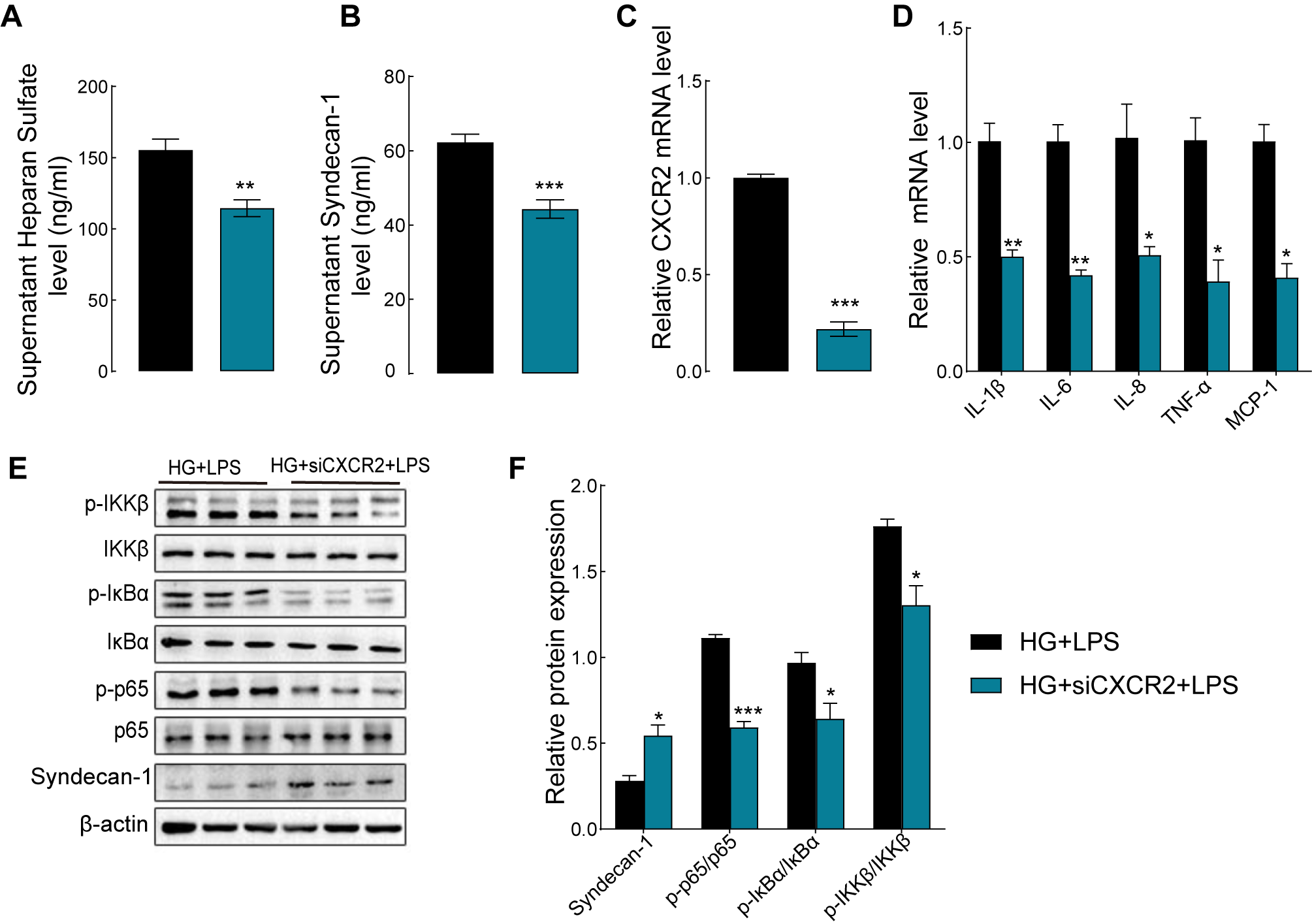

Supplement: Supplementary file 4 — Additional file 4: Supplementary Fig. 4. CXCR2 silence and overexpression efficiency in GECs. The mRNA(A) and protein(B and C) level of CXCR2 after transfection with CXCR2 siRNA1, siRNA2, siRNA3 in GECs, universal negative control siRNA was used as a control. The mRNA(D) and protein(E and F) level of CXCR2 transfection with pcDNA3.1-CXCR2 in GECs, the universal negative control siRNA or the pcDNA3.1 empty vector was used as a control. Results are expressed as mean ± SEM; ***P < 0.001 vs. control group; nsP > 0.05. [file 12964_2024_1565_MOESM4_ESM.docx]
