# Supplementary material for: Endothelial CXCR2 deficiency attenuates renal inflammation and glycocalyx shedding through NF-κB signaling in diabetic kidney disease
Source: Cell Commun Signal. 2024 Mar 25;22:191. doi: 10.1186/s12964-024-01565-2 (PMC10964613; doi:10.1186/s12964-024-01565-2)

**Supplementary Fig 7 The inflammation in CXCR2 overexpression GECs. (A)**The mRNA level of CXCR2 in four groups was detected. And we also tested the TNF-α, IL-1β, IL-6, and MCP-1 mRNA levels .(n=3)**(B)**. *CXCL1*(C) and *CXCL8* (D) mRNA level in GECs of four groups. Results are expressed as mean ± SEM; *P< 0.05, **P< 0.01, ***P< 0.001 vs. control group; ^&&&^P< 0.01 vs. HG group; ^###^P < 0.001 vs. HG+pcDNA3.1-CXCR2group; HG, high glucose; ^ns^P>0.05


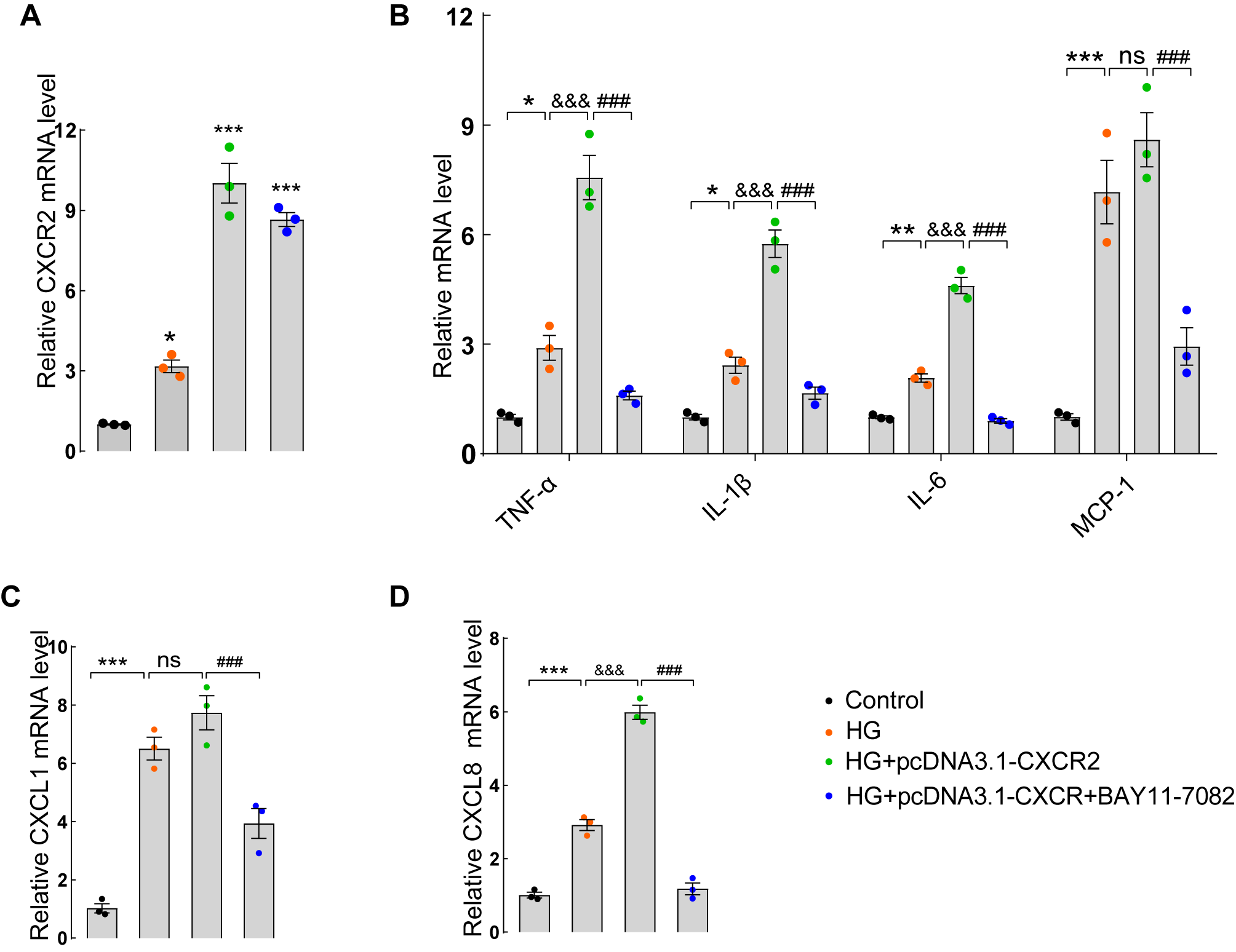

Supplement: Supplementary file 5 — Additional file 5: Supplementary Fig. 5. The inflammation in CXCR2 knockout GECs. The qPCR assay employed to quantify the level of CXCR2 in four groups. In the two siCXCR2 group, the expression were minimal, demonstrating that CXCR2 was successfully silenced (A). TNF-α, IL-1β, IL-6, and MCP-1 levels in four groups of GECs were also tested by qPCR (B). Elisa was used to test the levels of CXCL1(C) and CXCL8(D) in supernatant. Representative images were shown; Results are expressed as mean ± SEM;*P < 0.05, **P < 0.01, ***P < 0.001 vs. control group; &&P < 0.01,&&&P < 0.001 vs. HG group; ##P < 0.01, ###P < 0.001 vs. HG + SiCXCR2 group; HG, high glucose. [file 12964_2024_1565_MOESM5_ESM.docx]
