# Supplementary material for: Endothelial CXCR2 deficiency attenuates renal inflammation and glycocalyx shedding through NF-κB signaling in diabetic kidney disease
Source: Cell Commun Signal. 2024 Mar 25;22:191. doi: 10.1186/s12964-024-01565-2 (PMC10964613; doi:10.1186/s12964-024-01565-2)

**Supplementary Fig3. Renal inflammatory response in four groups of mice.** Immunohistochemistry was used to evaluate the infiltration of macrophages (F4/80) **(A)** and neutrophils (MPO) in the kidneys of mice **(B)**. Positive area of F4/80 **(C)** and MPO **(D**) (x400, Scale bar=50μm, n=3) were tested. And qPCR experiments was used to quantify *cxcr2* mRNA expression in four groups of mice(E). **(F)**The mRNA levels of *(MCP-1 (Ccl2), Ccl5, Cxcl1 and Cxcl2* in the glomeruli of mice were detected (n=3). ELISA was used to measure the levels of TNF-α **(G**), IL-1β **(H**), IL-6 (**I**), and IL-18 **(J)** in the peripheral blood serum of the four groups of mice (n=8). ImageJ was used for quantitative analysis of the positive staining area. Representative images were shown; Results are expressed as mean ± SEM ; ^**^P< 0.01, ^***^P< 0.001vs. CXCR2^L/L^ group; ^#^P < 0.05, ^##^P < 0.01, ^###^P < 0.001 vs. DKD-CXCR2^L/L^ group. ^&&&^P<0.001, CXCR2^eCKO^ group vs. CXCR2^L/L^ group.


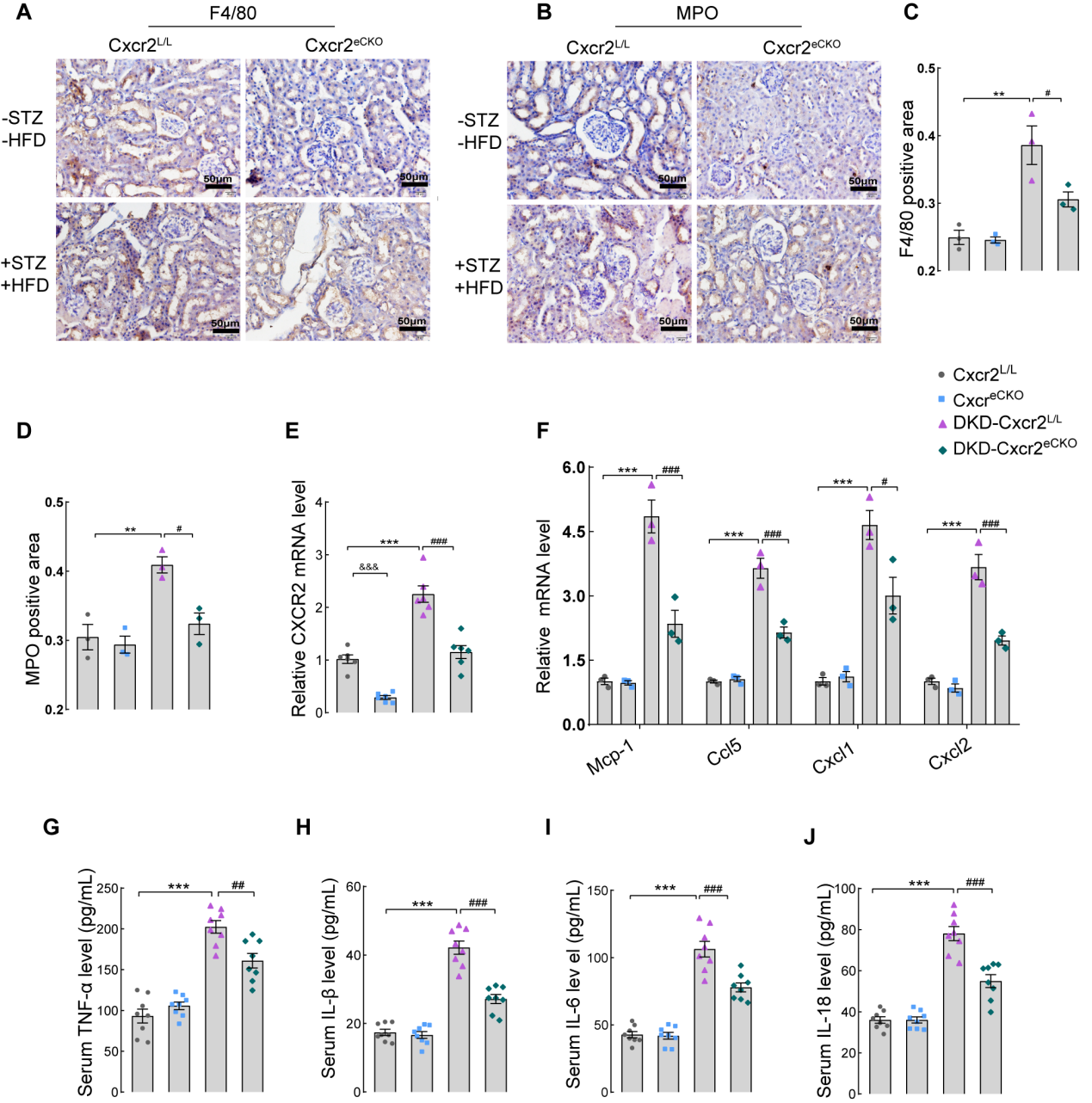

Supplement: Supplementary file 6 — Additional file 6: Supplementary Fig. 6. The levels of inflammatory factors and glycocalyx shedding in HG + LPS group and HG + LPS + siCXCR2 group. ELISA was used to measure the levels of heparan sulfate (A) and syndecan-1(B) in the supernatant in GECs of two groups. (C) The CXCR2 mRNA level in two groups. (D) qPCR experiments was used to quantify inflammatory factors mRNA levels. (E and F) The protein levels of syndecan-1, p-IKKβ, IKKβ, p-IκBα, IκBα, p-NF-κBp65, and NF-κB p65 were detected by western blotting. β-Actin was used as an internal reference control (n = 3). Results are expressed as mean ± SEM; *P < 0.05,**P < 0.01,***P < 0.001, HG + siCXCR2 + LPS vs. HG + LPS group; the universal negative control siRNA was used as a control. [file 12964_2024_1565_MOESM6_ESM.docx]
