# Supplementary material for: Endothelial CXCR2 deficiency attenuates renal inflammation and glycocalyx shedding through NF-κB signaling in diabetic kidney disease
Source: Cell Commun Signal. 2024 Mar 25;22:191. doi: 10.1186/s12964-024-01565-2 (PMC10964613; doi:10.1186/s12964-024-01565-2)

**Supplementary Fig4. CXCR2 silence and overexpression efficiency in GECs*.*** The mRNA**(A)** and protein**(B and C)** level of CXCR2 after transfection with CXCR2 siRNA1, siRNA2, siRNA3 in GECs, universal negative control siRNA was used as a control.The mRNA**(D)** and protein**(E and F)** level of CXCR2 transfection with pcDNA3.1-CXCR2 in GECs, the universal negative control siRNA or the pcDNA3.1 empty vector was used as a control. Results are expressed as mean ± SEM; ***P< 0.001 vs. control group; ^ns^P>0.05.


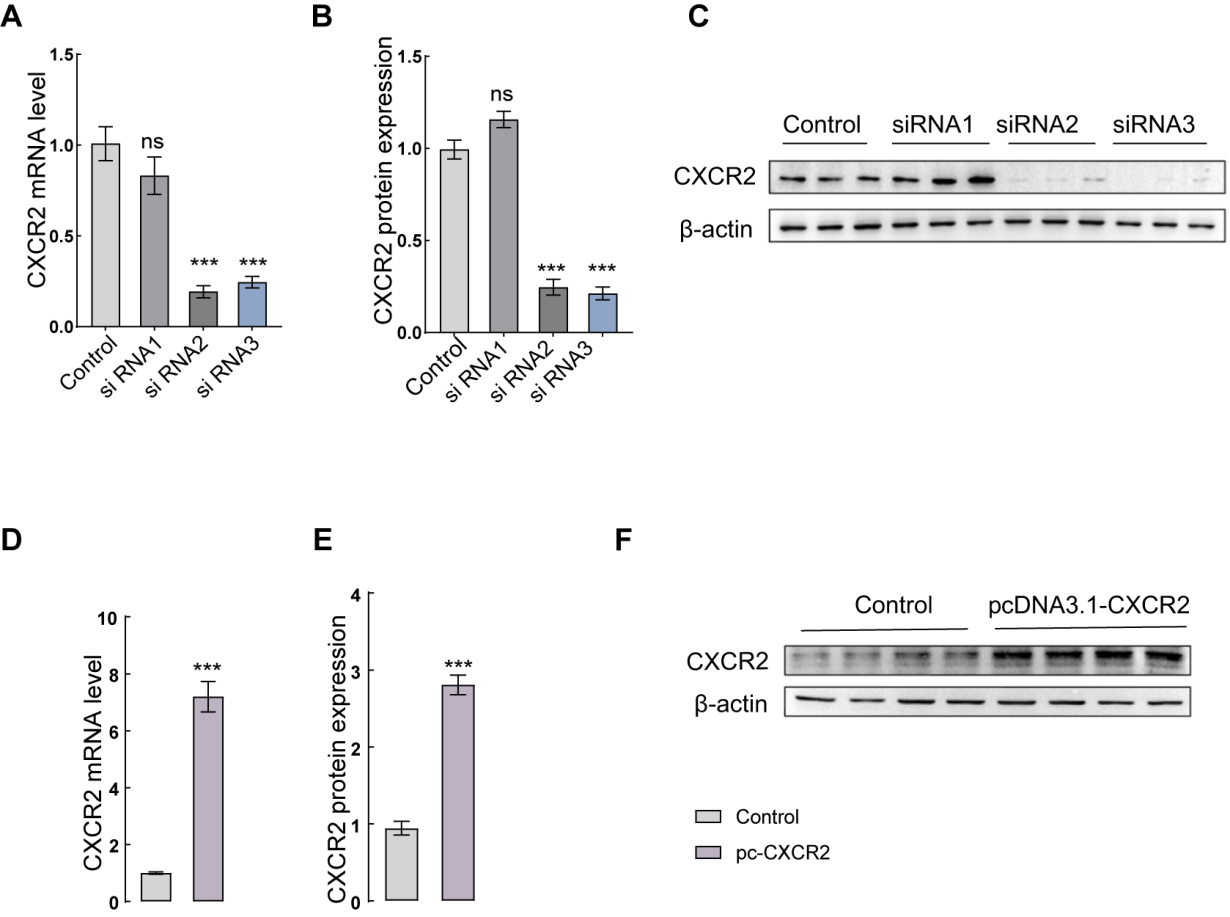

Supplement: Supplementary file 7 — Additional file 7: Supplementary Fig. 7. The inflammation in CXCR2 overexpression GECs. (A) The mRNA level of CXCR2 in four groups was detected. And we also tested the TNF-α, IL-1β, IL-6, and MCP-1 mRNA levels .(n = 3)(B). CXCL1(C) and CXCL8 (D) mRNA level in GECs of four groups. Results are expressed as mean ± SEM; *P < 0.05, **P < 0.01, ***P < 0.001 vs. control group; &&&P < 0.01 vs. HG group; ###P < 0.001 vs. HG + pcDNA3.1-CXCR2group; HG, high glucose; nsP > 0.05. [file 12964_2024_1565_MOESM7_ESM.docx]
