# Supplementary material for: Endothelial CXCR2 deficiency attenuates renal inflammation and glycocalyx shedding through NF-κB signaling in diabetic kidney disease
Source: Cell Commun Signal. 2024 Mar 25;22:191. doi: 10.1186/s12964-024-01565-2 (PMC10964613; doi:10.1186/s12964-024-01565-2)
Supplement: Supplementary file 8 — Additional file 8: Supplementary Table 1. The plasma and urine variables from different groups of humans. DKD, diabetic kidney disease; BMI, body mass index; FPG, fasting plasma glucose; HbA1c, glycosylated hemoglobin; BUN, blood urea nitrogen; Scr, Serum creatinine; eGFR, estimated glomerular filtration rate; UACR, urine albumin creatinine ratio; α1-MG, α1-microglobulin; RBP, retinol-binding protein; Compared with the Control group, **P<0.01. [file 12964_2024_1565_MOESM8_ESM.docx]

**Supplementary Table 1 . The plasma and urine variables from different groups of humans**. DKD, diabetic kidney disease; BMI, body mass index; FPG, fasting plasma glucose; HbA1c, glycosylated hemoglobin; BUN, blood urea nitrogen; Scr, Serum creatinine; eGFR, estimated glomerular filtration rate; UACR, urine albumin creatinine ratio; α1-MG, α1-microglobulin; RBP, retinol-binding protein; Compared with the Control group, ^**^*P*＜0.01.

| Parameters/groups | Control | DKD |
| --- | --- | --- |
| Gender (male，n %) | 28，56 | 26，52 |
| Age (years，$\overline{X}$±*s* ) | 55.18±9.75 | 52.70±8.03 |
| BMI (kg/m^2^，$\overline{X}$±*s*) | 25.03±2.90 | 24.62±3.31 |
| FPG [mmol/L， *M*（*Q*_1_， *Q*_3_）] | 4.82（4.44，5.38） | 9.37（7.92，12.11）^**^ |
| HbA1c (%，$\overline{X}$±*s*) | 5.27±0.51 | 9.19±2.71^**^ |
| BUN [mmol/L， *M*（*Q*_1_， *Q*_3_）] | 5.18（4.40，6.01） | 6.50（5.70，8.42） |
| Scr [umol/L， *M*（*Q*_1_， *Q*_3_）] | 55.45（48.30，65.95） | 84.10（56.60，152.80）^**^ |
| eGFR［ml·min⁻¹·（1.73 m²）⁻¹， *M*（*Q*_1_， *Q*_3_）］ | 102.50（96.00，111.00） | 88.50（63.00，96.00） |
| UACR [mg/g， *M*（*Q*_1_， *Q*_3_）] | 1.31（0.93，1.60） | 12.58（7.70，21.55）^**^ |
| α1-MG [ng/ml， *M*（*Q*_1_， *Q*_3_）] | 5.18（4.40，6.01） | 6.50（5.70，8.42）^**^ |
| RBP [ng/ml， *M*（*Q*_1_， *Q*_3_）] | 193.15（166.50，248.10） | 231.85（220.30，243.30）^**^ |
