# Supplementary material for: Endothelial CXCR2 deficiency attenuates renal inflammation and glycocalyx shedding through NF-κB signaling in diabetic kidney disease
Source: Cell Commun Signal. 2024 Mar 25;22:191. doi: 10.1186/s12964-024-01565-2 (PMC10964613; doi:10.1186/s12964-024-01565-2)
Supplement: Supplementary file 9 — Additional file 9: Supplementary Table 2. The CXCR2 siRNA sequences. [file 12964_2024_1565_MOESM9_ESM.docx]

**Supplementary Table 2. The CXCR2 siRNA sequences**

| **Gene Name** | Organisms |  | **Sequence ( 5' → 3' )** |
| --- | --- | --- | --- |
| siRNA1 | Homo sapiens | F | CCGUCUACUCAUCCAAUGUUATT |
|  |  | R | UAACAUUGGAUGAGUAGACGGTT |
| siRNA2 | Homo sapiens | F | GCUACUUGGUCAAAUUCAUTT |
|  |  | R | AUGAAUUUGACCAAGUAGCTT |
| siRNA3 | Homo sapiens | F | GCUAUACAUGGCUUGAUCAGCTT |
|  |  | R | GCUGAUCAAGCCAUGUAUAGCTT |
