# Supplementary material for: Endothelial CXCR2 deficiency attenuates renal inflammation and glycocalyx shedding through NF-κB signaling in diabetic kidney disease
Source: Cell Commun Signal. 2024 Mar 25;22:191. doi: 10.1186/s12964-024-01565-2 (PMC10964613; doi:10.1186/s12964-024-01565-2)
Supplement: Supplementary file 10 — Additional file 10: Supplementary Table 3. Primer sequences of Mus musculus used for qRT-PCR. [file 12964_2024_1565_MOESM10_ESM.docx]

**Supplementary Table 3.** Primer sequences of Mus musculus used for qRT-PCR

| **Gene Name** | Organisms |  | **Sequence ( 5' → 3' )** |
| --- | --- | --- | --- |
| Ccl2 | Mus musculus | F | TTAAAAACCTGGATCGGAACCAA |
|  |  | R | GCATTAGCTTCAGATTTACGGGT |
| Ccl5 | Mus musculus | F | GCTGCTTTGCCTACCTCTCC |
|  |  | R | TCGAGTGACAAACACGACTGC |
| Cxcl1 | Mus musculus | F | CTGGGATTCACCTCAAGAACATC |
|  |  | R | CAGGGTCAAGGCAAGCCTC |
| Cxcl2 | Mus musculus | F | CCAACCACCAGGCTACAGG |
|  |  | R | GCGTCACACTCAAGCTCTG |
| Cxcr2 | Mus musculus | F | CACAAACAGCGTCGTAGAACT |
|  |  | R | ACCAAGGAGTTCCCCACAAG |
| β-actin | Mus musculus | F | TGTTACCAACTGGGACGACA |
|  |  | R | CTGGGTCATCTTTTCACGGT |
