# Supplementary material for: Endothelial CXCR2 deficiency attenuates renal inflammation and glycocalyx shedding through NF-κB signaling in diabetic kidney disease
Source: Cell Commun Signal. 2024 Mar 25;22:191. doi: 10.1186/s12964-024-01565-2 (PMC10964613; doi:10.1186/s12964-024-01565-2)
Supplement: Supplementary file 11 — Additional file 11: Supplementary Table 4. Primer sequences of Homo sapiens used for qRT-PCR [file 12964_2024_1565_MOESM11_ESM.docx]

**Supplementary Table 4.** Primer sequences of Homo sapiens used for qRT-PCR

| **Gene Name** |  |  | **Sequence ( 5' → 3' )** |
| --- | --- | --- | --- |
| TNF-α | Homo sapiens | F | TCTCTAATCAGCCCTCTG |
|  |  | R | GGGTTTGCTACAACATGG |
| IL-1β | Homo sapiens | F | TGGCTTATTACAGTGGCAATGAG |
|  |  | R | TAGTGGTGGTCGGAGATTCG |
| IL-6 | Homo sapiens | F | ACTCACCTCTTCAGAACGAATTG |
|  |  | R | CCATCTTTGGAAGGTTCAGGTTG |
| MCP-1 | Homo sapiens | F | CTCGCTCAGCCAGATGCAAT |
|  |  | R | CACTTGCTGCTGGTGATTCTTCT |
| CXCL1 | Homo sapiens | F | GCTTGCCTCAATCCTGCATC |
|  |  | R | AGTTGGATTTGTCACTGTTCAGC |
| CXCL8 | Homo sapiens | F | ACTGAGAGTGATTGAGAGTGGAC |
|  |  | R | AACCCTCTGCACCCAGTTTTC |
| CXCR2 | Homo sapiens | F | CTCTAAGACCTCCTGCCTAAGT |
|  |  | R | CATTGACACTGAGACCAAGAAGAA |
| E-selectin | Homo sapiens | F | TGCAAGTTCGCCTGTCCTGA |
|  |  | R | GAGGGAGAGTCCAGCAGCAG |
| MMP-9 | Homo sapiens | F | TCTGTGCGTTATGGTTCA |
|  |  | R | CAGGAAGACACTTGGTTATC |
| MMP-2 | Homo sapiens | F | AGATTGACGCTGTGTATGA |
|  |  | R | ATGTATGTCTTCTTGTTCTTACTC |
| HPSE | Homo sapiens | F | TCCTGCGTACCTGAGGTTTG |
|  |  | R | CCATTCCAACCGTAACTTCTCCT |
| HYAL1 | Homo sapiens | F | CGATATGGCCCAAGGCTTTAG |
|  |  | R | ACCACATCGAAGACACTGACAT |
| β-ACTIN | Homo sapiens | F | CATGTACGTTGCTATCCAGGC |
|  |  | R | CTCCTTAATGTCACGCACGAT |
